# Effects of lipid-based nutrient supplements and infant and young child feeding counseling with or without improved water, sanitation, and hygiene (WASH) on anemia and micronutrient status: results from 2 cluster-randomized trials in Kenya and Bangladesh

**DOI:** 10.1093/ajcn/nqy239

**Published:** 2019-01-09

**Authors:** Christine P Stewart, Kathryn G Dewey, Audrie Lin, Amy J Pickering, Kendra A Byrd, Kaniz Jannat, Shahjahan Ali, Gouthami Rao, Holly N Dentz, Marion Kiprotich, Charles D Arnold, Benjamin F Arnold, Lindsay H Allen, Setareh Shahab-Ferdows, Ayse Ercumen, Jessica A Grembi, Abu Mohd Naser, Mahbubur Rahman, Leanne Unicomb, John M Colford, Stephen P Luby, Clair Null

**Affiliations:** 1 Department of Nutrition, University of California, Davis, Davis, CA; 2 Division of Epidemiology and Biostatistics, University of California, Berkeley, Berkeley, CA; 3 Department of Civil and Environmental Engineering; 4 Division of Infectious Diseases and Geographic Medicine, Stanford University, Stanford, CA; 5 International Center for Diarrheal Disease Research, Bangladesh, Dhaka, Bangladesh; 6 Innovations for Poverty Action, Nairobi, Kenya; 7 USDA, Agricultural Research Service, Western Human Nutrition Research Center, Davis, CA

**Keywords:** lipid-based nutrient supplement, water, sanitation, hygiene, anemia, micronutrients, children

## Abstract

**Background:**

Anemia in young children is a global health problem. Risk factors include poor nutrient intake and poor water quality, sanitation, or hygiene.

**Objective:**

We evaluated the effects of water quality, sanitation, handwashing, and nutrition interventions on micronutrient status and anemia among children in rural Kenya and Bangladesh.

**Design:**

We nested substudies within 2 cluster-randomized controlled trials enrolling pregnant women and following their children for 2 y. These substudies included 4 groups: water, sanitation, and handwashing (WSH); nutrition (N), including lipid-based nutrient supplements (LNSs; ages 6–24 mo) and infant and young child feeding (IYCF) counseling; WSH+N; and control. Hemoglobin and micronutrient biomarkers were measured after 2 y of intervention and compared between groups using generalized linear models with robust SEs.

**Results:**

In Kenya, 699 children were assessed at a mean ± SD age of 22.1 ± 1.8 mo, and in Bangladesh 1470 participants were measured at a mean ± SD age of 28.0 ± 1.9 mo. The control group anemia prevalences were 48.8% in Kenya and 17.4% in Bangladesh. There was a lower prevalence of anemia in the 2 N intervention groups in both Kenya [N: 36.2%; prevalence ratio (PR): 0.74; 95% CI: 0.58, 0.94; WSH+N: 27.3%; PR: 0.56; 95% CI: 0.42, 0.75] and Bangladesh (N: 8.7%; PR: 0.50; 95% CI: 0.32, 0.78; WSH+N: 7.9%, PR: 0.46; 95% CI: 0.29, 0.73). In both trials, the 2 N groups also had significantly lower prevalences of iron deficiency, iron deficiency anemia, and low vitamin B-12 and, in Kenya, a lower prevalence of folate and vitamin A deficiencies. In Bangladesh, the WSH group had a lower prevalence of anemia (12.8%; PR: 0.74; 95% CI: 0.54, 1.00) than the control group, whereas in Kenya, the WSH+N group had a lower prevalence of anemia than did the N group (PR: 0.75; 95% CI: 0.53, 1.07), but this was not significant (*P* = 0.102).

**Conclusions:**

IYCF counseling with LNSs reduced the risks of anemia, iron deficiency, and low vitamin B-12. Effects on folate and vitamin A varied between studies. Improvements in WSH also reduced the risk of anemia in Bangladesh but did not provide added benefit over the nutrition-specific intervention.

These trials were registered at clinicaltrials.gov as NCT01590095 (Bangladesh) and NCT01704105 (Kenya).

## Introduction

Approximately 40% of young children in low- and middle-income countries suffer from anemia, a problem that can lead to fatigue, developmental delays, poor school performance, and an increased risk of mortality (1). Anemia is caused by micronutrient deficiencies, infectious diseases, and inherited hemoglobin disorders, but the relative importance of these causes varies substantially between countries (2). The WHO estimates that ∼40% of cases of anemia in preschool children could be eliminated with iron supplementation (1), but deficiencies of vitamin B-12, folate, riboflavin, and vitamin A are also potentially important causal factors (3). Soil-transmitted helminths, such as hookworm or *Trichuris trichiura* infection, can cause blood loss leading to anemia (4). Chronic inflammation is also associated with anemia, partially through a downregulation of iron absorption (5), and may be caused by diarrheal disease or environmental enteric dysfunction (6). Malaria is also a significant contributor to anemia, particularly in sub-Saharan Africa (2). In South Asia, nearly 97 million children aged <5 y are estimated to have anemia, approximately half of all children in the region, and in Africa, the prevalence is even higher at 62% (1).

Although there is strong evidence that micronutrient supplementation or fortification is an effective strategy for anemia prevention (7), there is less evidence on the impacts of water, sanitation, and hygiene (WASH) practices. It is plausible that WASH interventions could be efficacious in reducing anemia by preventing helminth, especially hookworm, infections, reducing diarrhea, and reducing chronic inflammation (6). Improved sanitation and hygiene have also been proposed as interventions to reduce environmental enteric dysfunction, a condition characterized by subclinical immune activation, gut inflammation, villous atrophy, and permeability, which, in turn, could reduce the risk of anemia (6). Observational studies have presented some evidence of an association between poor sanitation and anemia (2, 8); however, there is no evidence from intervention trials.

The WASH Benefits Study evaluated the effects of water, sanitation, handwashing, and nutrition interventions delivered alone and in combination in 2 highly comparable trials in Kenya and Bangladesh (NCT01590095 in Bangladesh; NCT01704105 in Kenya). The primary outcomes of the trial included length-for-age *z* score and diarrhea, and secondary outcomes included child development, environmental enteric dysfunction, and other measures of child anthropometric status. We recently reported that there were significant effects on growth in the 2 nutrition (N) arms in both trials, but none in the water, sanitation, and handwashing (WSH) arms (9, 10). The effects on diarrhea varied between the trials, with significantly lower prevalences across all arms in Bangladesh but no effects in Kenya. This article focuses on substudies performed within 4 arms of both trials: *1*) control; *2*) N, comprising infant and young child feeding (IYCF) behavior change messaging plus lipid-based nutrient supplements (LNSs); *3*) WSH; and *4*) WSH+N. These substudies evaluated 2 hypotheses: *1*) improvements in N, WSH, or WSH+N would improve biomarkers of micronutrient status and reduce the risk of anemia in young children and *2*) the combination of WSH+N would improve biomarkers of micronutrient status and reduce the risk of anemia more than either WSH or N alone.

## Methods

Detailed descriptions of the 2 trials have been previously published (9–13).

### Kenya study site and interventions

The trial was implemented in 3 counties in western Kenya—Kakamega, Bungoma, and Vihiga. Geographically matched village clusters were block-randomized into 1 of 8 study arms: chlorine treatment of drinking water (W); improved sanitation limiting exposure to feces (S); handwashing with soap (H); combined WSH; IYCF counseling plus small-quantity LNSs (N); combined WSH+N; active control in which children were visited by a health promoter monthly; and passive control with no intervention household visits.

Villages were eligible for inclusion into the trial if they were rural, with most of the population reliant on unimproved sanitation facilities, without widespread access to chlorinated water, and without any active water, sanitation, handwashing, or nutrition programs. Households were eligible if there was a woman in her second or third trimester of pregnancy who planned to live in the community for ≥2 y and who could speak Kiswahili, Luhya, or English. Intervention clusters were formed by including a minimum of 6 eligible pregnant women residing in ≤3 neighboring villages. There were no buffer zones between clusters due to geographic constraints.

Community health promoters (CHPs) were nominated by women in the community and trained to provide the intervention-specific behavior change activities as well as instructions for provision of supplements or hardware use. In both the active control and intervention arms, they were trained to measure midupper arm circumference to identify and provide referrals for potential cases of severe acute malnutrition.

Each intervention package consisted of a comprehensive behavior change promotion program, including key messages; visual aids in the form of flip charts, posters, and reminder cue cards; interactive activities with songs, games, and pledges; and the distribution of arm-specific hardware, products, or supplements (10). CHPs were instructed to visit homes once per month throughout the trial, although visit frequency declined during the second year of the trial (10). In the active control arm, CHPs visited households at the same frequency as in the intervention arms.

In the N intervention arm, households received monthly rations of micronutrient-fortified, small-quantity LNSs (Nutriset) when children were between the ages of 6 and 24 mo. The composition of the LNSs in comparison to the Recommended Nutrient Intakes for children is shown in **Supplemental Table 1**. At the start of the trial, supplements were distributed by CHPs, whereas in the second year of the study, project staff provided the monthly rations. Supplements were provided for the index child plus any other age-eligible siblings in the household. Caregivers were instructed to mix one 10-g sachet into the child's complementary foods twice per day. Key messages focused on standard IYCF recommendations, including maternal dietary diversity during pregnancy and lactation, early initiation of breastfeeding, exclusive breastfeeding from age 0 to 6 mo and continued breastfeeding through 24 mo, timely introduction of complementary foods at 6 mo, dietary diversity, feeding frequency, and feeding during illness.

In the WSH intervention arm, CHPs advocated for a variety of behaviors to improve water quality, sanitation, and handwashing practices within the home. Specifically, they promoted treatment of drinking water with sodium hypochlorite using either chlorine dispensers installed at the point-of-collection in study villages or bottled chlorine provided directly to households. They also used chlorine test strips to spot-check household chlorine concentrations during monthly visits; results were used to improve counseling. Existing latrines were upgraded and improved by installing a plastic slab with a tight-fitting lid. Households with no latrine were provided with a new one. CHPs provided a “sani-scoop” with a paddle to remove animal and human fecal material from the yard surrounding the home and child potties for each child aged <3 y in the study compound. Households were also provided with 2 dual tippy-tap handwashing stations, one near the latrine and a second near the cooking area. Stations were designed to have 2 foot-pedal–operated jerry cans that could be tipped to dispense a small stream of either soapy water or rinse water (14). CHPs filled the soapy water dispenser with soap every 3 mo. In the WSH+N arm, all of the aforementioned activities, hardware, and supplements were provided.

Adherence to LNS recommendations was high (>90%) in the N arms, but there were no differences in complementary feeding practices between groups (10, 15). Adherence to the WSH interventions varied, but was generally lower than the LNS adherence (10).

### Bangladesh study site and interventions

The WASH Benefits Bangladesh Trial was implemented in subdistricts of the Gazipur, Kishoreganj, Mymensingh, and Tangail districts. The study area was selected because it had low groundwater iron and arsenic according to data obtained from the Department of Public Health Engineering/British Geological Survey/Department for International Development National Hydrochemical Survey and a survey conducted before study initiation (16, 17) (**Supplemental Figure 1**); and it had no major water, sanitation, or focused nutrition programs underway or planned. Clusters were randomized by geographic blocks into 1 of 7 study arms: chlorine treatment of drinking water (W); improved sanitation limiting exposure to feces (S); handwashing with soap (H); combined WSH; IYCF counseling plus small-quantity LNSs (N); combined WSH+N; or a control group that received no intervention. In contrast to Kenya, there was no active control arm.

Households with a pregnant woman were invited to participate in the trial if they planned to reside in the study communities for ≥2 y. The subsequent child or children, in the case of multiple births, born to those women were considered the study index children. Intervention clusters were formed with 8 eligible pregnant women in nearby proximity with a minimum buffer zone of 1 km between clusters to minimize intervention spillover effects.

Local community members were recruited to serve as CHPs for the trial. Individuals who lived within walking distance of an intervention cluster, who had completed ≥8 y of formal education, and who passed a written and oral exam were considered eligible. CHPs attended an arm-specific training session at the start of the trial plus quarterly refresher trainings. Training sessions focused on the behavioral recommendations and intervention hardware usage, as well as communication and active listening techniques, and approaches for collaborative problem solving with the enrolled mothers. CHPs were instructed to visit study households at least once per week in the first 6 mo and fortnightly thereafter, although in practice, visits averaged 6 times/mo throughout the trial (9). Each intervention package consisted of arm-specific behavioral recommendations, hardware, or supplements delivered by the health promoter. There were no promoter visits or other intervention activities in the control-group households.

The N intervention was similar to that described for Kenya. LNSs were delivered monthly by CHPs and provided for the index child only. IYCF recommendations were adapted from those developed by the Alive and Thrive program in Bangladesh (18).

In the WSH intervention arm, households were provided a 10-L vessel with a lid, a tap, and a regular supply of 33-mg sodium dichloroisocyanurate (NaDCC) tablets (Medentech) to treat and safely store their drinking water. Households were encouraged to fill the vessel, add 1 tablet, and wait 30 min before drinking the water. Households that did not have access to an improved latrine with a slab and functional water seal in their compound were provided either a new latrine or improvements to an existing latrine. Households were also provided a scoop that could be used to clean the home environment and a child potty for all children aged <3 y for the safe disposal of child feces. Handwashing practices were promoted and supported by providing 2 handwashing stations, one placed near the latrine and the second placed near the kitchen. Each station contained a water reservoir with a tap, a basin to collect the rinse water, and a soapy water bottle. CHPs provided a regular supply of detergent for making the soapy water. The WSH+N intervention group received all of the aforementioned behavioral interventions, hardware, and products. The adherence to LNS supplementation and all behavioral recommendations was high across all groups throughout the trial (9) and dietary diversity was significantly greater in the 2 N arms (19).

### Randomization and masking

Interventions were randomly assigned at the cluster level with the use of codes generated by an off-site investigator independent of the data collection team via a random-number generator. Groups of adjacent clusters were block-randomized into the 6 intervention arms or a double-sized passive control arm (in Bangladesh) or double-sized active control arm and single-sized passive control arm (in Kenya). Participants were informed of their group assignment after the baseline survey. Masking participants was not possible due to the nature of the interventions. Although data collectors were not directly informed of the random assignment, they may have inferred the intervention group through observation of materials within the home during subsequent household visits. Masked technicians completed the laboratory analyses.

In both trials, substudy clusters were selected from the N, WSH, WSH+N, and control arms (active in Kenya and passive in Bangladesh). Clusters were selected based on the logistical feasibility of the preservation and collection of biological specimens, as well as their transport to the central laboratory. The primary objective of the substudies was to assess biomarkers of environmental enteric dysfunction, which will be reported elsewhere. Index households with live-born infants residing in selected clusters were invited to participate in the substudy activities. Exclusion criteria included symptoms of moderate to severe dehydration on the day of the survey, as follows: *1*) restless, irritable; *2*) sunken eyes; *3*) drinks eagerly, thirsty; and *4*) pinched skin returns to normal position slowly or the child being listless or unable to perform their normal activities.

### Outcomes

In both trials, after consent and enrollment, enumerators administered a baseline questionnaire asking respondents to report on their household characteristics, animal and other asset ownership, food insecurity status using the Household Food Insecurity Access Scale (20) in Bangladesh and the Household Hunger Scale (21) in Kenya, and WASH conditions within the home. In addition to the 2 follow-up visits that all study children received, children in the substudies were visited at 3 additional times for data and biological sample collection. Anemia and micronutrient status were measured at the third of these follow-up visits. In Kenya, this occurred ∼2 y after the start of intervention activities. In Bangladesh, fieldwork was delayed by ∼4 mo at the third follow-up point due to civil unrest, which also caused inconsistency in the timing of follow-up visits in some clusters.

Substudy visits were conducted within the home in Bangladesh and at a central site in the community in Kenya. For each child, a maximum of 7.7 mL blood was collected by a trained phlebotomist via venipuncture into a Sarstedt monovette serum collection tube and a Sarstedt monovette lithium heparin trace element–free plasma collection tube. Hemoglobin concentrations were measured in venous whole blood at the point of collection using a portable spectrophotometer (Hemocue 301) that was regularly checked using standardized quality-control procedures. In Kenya, malaria was tested using rapid diagnostic test kits (Alere; SD Bioline Malaria Ag P.f/P.f/P.v) at the time of collection. In Kenya, blood samples were placed on ice packs, centrifuged at 3500 rpm for 15 min at ambient temperature within a mean of 1 h of collection, and then plasma and serum aliquots were placed on ice packs in a cooler where they remained for ∼2.5 h until they could be transferred to a −20°C freezer. Within ∼2 wk of collection, samples were subsequently transferred to −80°C freezers at the Kenya Medical Research Institute (KEMRI) in Nairobi for long-term storage. In Bangladesh, blood samples were placed on ice, transported to the project laboratory, centrifuged at 3500 rpm for 15 min at ambient temperature, and stored in a −80°C freezer. The mean time from blood collection to centrifugation was 8.5 h.

Specimens were shipped on dry ice to collaborating laboratories for analysis. Serum retinol-binding protein (RBP), ferritin, soluble transferrin receptor (sTfR), α-1 acid glycoprotein (AGP), and C-reactive protein (CRP) were measured using ELISA methods (22) (VitMin Lab). Quality-control material from the CDC and Biorad Liquicheck Controls were used to ensure the accuracy and precision of the analysis. A manufacturer lot problem with the AGP assay affected approximately half the Bangladesh samples and there was insufficient sample volume available to rerun the samples, so those results are not presented. Hepcidin was measured using a commercially available ELISA kit and controls (Peninsula Laboratories) following the manufacturer's protocol at the International Center for Diarrheal Disease Research, Bangladesh, or KEMRI laboratories. Serum vitamin B-12 and folate concentrations were measured using chemiluminescence on a Roche e411 with Roche B-12 and folate III reagents, standards, and controls to ensure accuracy and precision of the assay at the USDA Western Human Nutrition Research Center in Davis, California. External quality control was monitored through the CDC Vital-External Quality Assurance Program. Intra-assay CVs from replicate measures for all micronutrient biomarkers, CRP, and AGP were <10% in Bangladesh and <12% in Kenya. The highest CV was found in the hepcidin assay in Kenya (11.5%), which was driven by 7 samples with a large variation. Removing those subjects from the analysis did not substantially change the interpretation of our results and so we have elected to keep them in the analysis. In Bangladesh, whole-blood samples were tested for thalassemia and hemoglobin E (HbE) traits using electrophoresis at Dhaka Shishu Hospital. In Kenya, DNA was extracted from packed blood cell samples and tested for sickle cell trait and α-thalassemia by polymerase chain reaction using proprietary kits (Qiagen) (23, 24) at KEMRI/Wellcome Trust laboratories in Kilifi, Kenya.

The sample size for the substudy was based on detecting a difference of ≥0.26 SDs in standardized log environmental enteric dysfunction biomarkers (reported separately) between any intervention arm and the control arm assuming 80% power, a 2-sided type 1 error of 5%, and an intracluster correlation coefficient of 0.15, resulting in a sample size of *n* = 375 children/group, assuming 54 clusters/arm and 7 children/cluster (https://osf.io/qa43y/). Using this number, we estimated that we would be able to detect a mean difference in hemoglobin between groups of 3.4–3.9 g/L or a difference in the prevalence of anemia of 11.3–11.9 percentage points based on an assumed mean hemoglobin of 103 g/L and prevalence of anemia of 47–85%, based on previously published data from children in nearby study areas (25, 26). The intracluster correlation was estimated to be between 0.6 and 0.14 for hemoglobin and 0.07 and 0.10 for anemia, based on data from 3 large studies in India, Indonesia, and Vietnam (27–29).

### Ethics

Mothers provided written informed consent for themselves and their infants. The study protocol was reviewed and approved by the Committee for the Protection of Human Subjects at the University of California, Berkeley, the Institutional Review Board at Stanford University, the Ethical Review Committee at the International Center for Diarrheal Disease Research, Bangladesh, and the Scientific and Ethics Review Committee at the KEMRI. Children with hemoglobin <70 g/L or who tested positive for malaria with fever or sickle cell disease (HbSS) were referred for treatment.

### Statistical analysis

Detailed analysis plans were prespecified and publicly posted (https://osf.io/dsrv2). Analyses were independently replicated by 2 investigators (CDA and KAB) using R version 3.5.0 (R Foundation for Statistical Computing) and Stata version 14 (StataCorp LLC). All investigators were blinded to group assignment until primary analyses were completed.

Hemoglobin concentrations were adjusted for altitude, and a hemoglobin cutoff of 110 g/L was used to define anemia (30). We further calculated the prevalence of mild (hemoglobin of 100–109 g/L), moderate (hemoglobin of 70–99 g/L), and severe (hemoglobin <70 g/L) anemia. Micronutrient deficiencies were defined in the following ways: iron deficiency as either ferritin <12 µg/L or sTfR >8.3 mg/L (22); vitamin A deficiency as RBP <0.83 µmol/L (31); folate deficiency as <10 nmol/L (32, 33); and vitamin B-12 deficiency as <150 pmol/L and depletion as 150–221 pmol/L (34). We also examined low hepcidin concentrations using a cutoff of 5.5 ng/mL (35). Further, high folate was categorized as >45.3 nmol/L (36). Elevated CRP (>5 mg/L) or AGP (>1 g/L; in Kenya only) was used to define inflammation (37).

We estimated the mean difference in each of the biomarkers between each intervention group and the control group using generalized linear models with robust SEs controlling for clustering at the study block level. Skewed outcomes were log-transformed for analysis. We also calculated the prevalence difference and prevalence ratio between groups using a linear probability model for the prevalence differences and a binomial distribution with a log link for the prevalence ratios, each with robust SEs at the study block level. Our primary inference was the unadjusted between-group differences in these parameters. The primary models were not adjusted for inflammation because this might be on the causal pathway between the interventions and the outcomes.

In secondary analyses, we additionally adjusted for prespecified baseline covariates that may be potentially associated with the outcomes, including child age, sex, birth order, maternal age, height, educational level, household food insecurity category, number of children aged <18 y in the household, number of individuals living in the compound, distance to the primary water source, housing materials, household assets, animal ownership, malaria infection, and thalassemia, sickle cell, or HbE traits. Further, we also considered the potential effects of inflammation on the interpretation of the deficiency prevalence estimates. Ferritin, sTfR, and RBP have been found to be sensitive to the acute-phase response (37, 38). We used the regression method proposed by the Biomarkers Reflecting Inflammation and Nutritional Determinants of Anemia research group (39–41). Finally, we considered the potential for interaction between the intervention group and child age, hemoglobinopathy trait, sex, and household food insecurity status.

In additional analyses, and to examine the potential for bias due to losses to follow-up, we examined whether there were differences in the characteristics of those with missing data compared with those who were included in this analysis. We conducted an inverse probability of censoring–weighted (IPCW) analysis that reweighted the analysis population to reflect the original enrolled population (42, 43). The reference population consisted of all children selected for the substudy, excluding known pregnancy losses. Baseline measurements including maternal parity, age, education, household hunger score, number of children aged <18 y in the household, number of people living in the compound, distance to primary water source, improved primary water source, roofing quality, asset index score, animal ownership, and timing of measurement in the cluster were used to predict missingness. Missingness mechanism parameters were estimated using logistic regression, and targeted maximum likelihood was used for treatment effect inference. In post hoc analysis, we additionally examined the micronutrient concentrations by study month in Bangladesh to examine seasonal patterns.

## Results

### Sample characteristics

**FIGURE 1:**
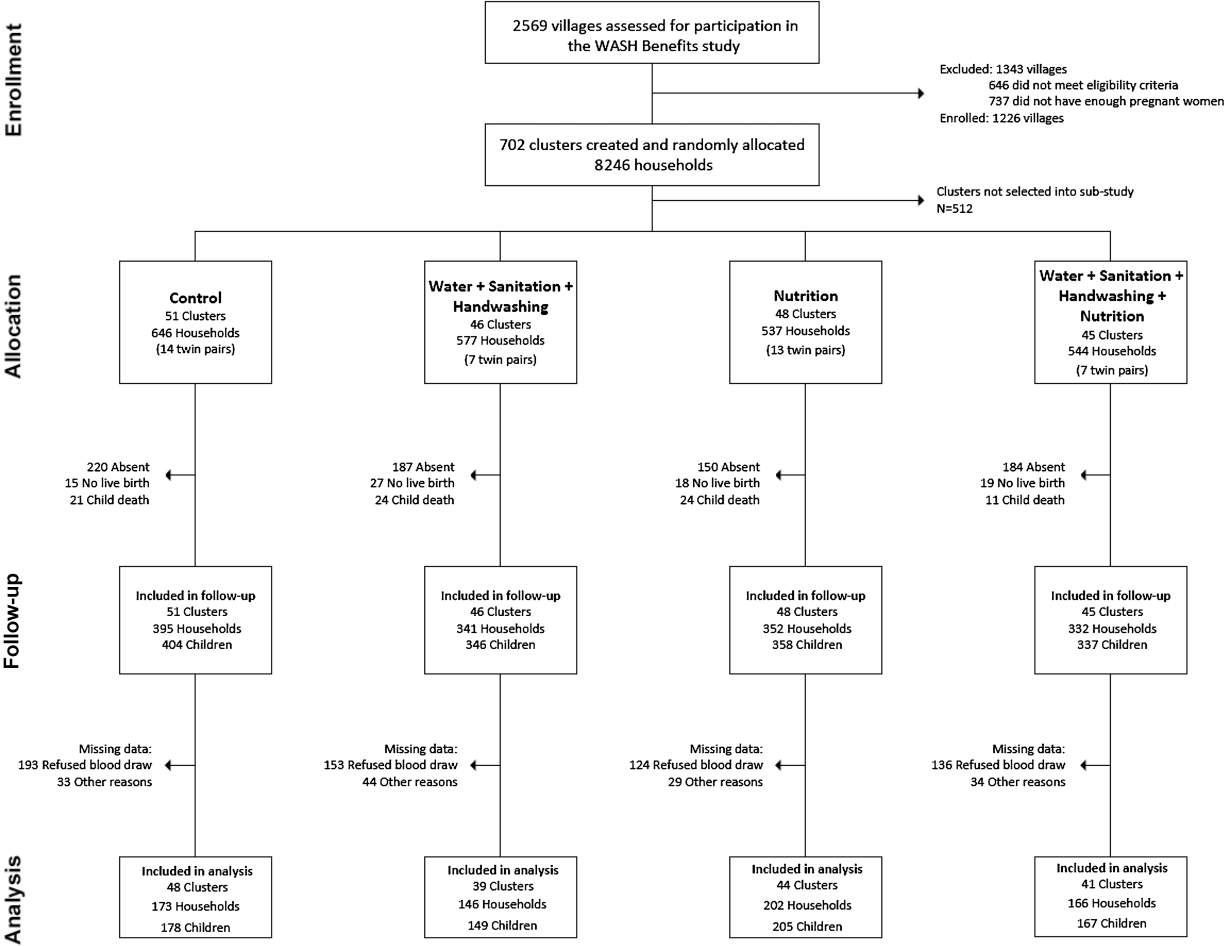
Summary of participant enrollment, random assignment, retention, and analysis in the WASH Benefits Kenya Study. WASH, water, sanitation, and hygiene.

In the Kenya trial, households were enrolled between November 2012 and May 2014 and the substudy follow-up occurred from July 2015 to April 2016. A total of 190 clusters with 2304 households were included in the substudy (**Figure 1**). Losses to follow-up were due to no live birth (*n* = 75), child death (*n* = 80), or not attending the study visit (*n* = 741). Among those who attended the study visit, an additional 608 refused to provide a blood sample and 142 had missing data due to insufficient blood volume collected or other reasons. Follow-up rates were similar across groups. Enrollment characteristics were generally balanced (**Table 1**). Mothers had a mean age of ∼26 y, approximately one-quarter were primiparous, and slightly less than half had completed at least primary schooling. The majority of households had access to an improved source of drinking water, but <15% reported treating their drinking water with chlorine. Latrine access was high (>80%), but improved latrines were rare (<20%). Open defecation was uncommon among adults, but common among children aged <3 y (>75%). The prevalence of moderate to severe hunger was just over 10%. Individuals who were included in the analysis sample were similar in terms of most characteristics to those with missing data and those in the main study cohort (**Supplemental Table 2**). The only notable difference was that those who were included in the analysis sample were less likely to be firstborn children (19.1%) compared with those in the substudy with missing data (26.7%) or those in the rest of the main study sample (24.0%). Adherence to LNS supplementation was high (>94% of expected sachets consumed) among age-eligible children at midline and endline (**Supplemental Table 3**).

**TABLE 1:** Enrollment characteristics by intervention group in the WASH Benefits Kenya Trial^1^

	Group			
	Active control	WSH	N	WSH+N
Households, *n*	632	550	521	525
Maternal				
Age, y	25.6 ± 6.2	25.7 ± 6.0	25.9 ± 6.1	25.8 ± 6.3
Height, cm	160.5 ± 5.8	160.1 ± 5.9	160.1 ± 6.0	160.3 ± 5.9
Primiparous, %	25.6	21.3	24.8	25.8
Completed at least primary education, %	45.3	46.0	49.5	50.3
Paternal, %				
Completed at least primary education	60.5	59.5	61.4	62.7
Works in agriculture	45.1	46.3	46.6	42.1
Household				
People per compound, *n*	8.4 ± 5.6	8.4 ± 5.3	8.8 ± 7.1	8.8 ± 5.9
Children aged <18 y in the household, *n*	2.8 ± 1.9	2.7 ± 1.9	2.8 ± 2.0	2.9 ± 2.0
Has electricity, %	4.3	6.9	6.7	6.3
Has a cement floor, %	5.1	5.3	5.0	6.1
Has an iron roof, %	59.7	59.3	65.5	64.6
Drinking water				
Walking time to primary water source, min	10.0 ± 10.6	10.2 ± 10.9	10.6 ± 11.1	10.0 ± 11.3
Primary drinking water source is improved,^2^ %	77.5	69.6	69.8	79.1
Reported treating currently stored water, %	11.3	12.6	6.9	13.5
Sanitation, %				
Own any latrine	81.6	83.8	84.1	86.8
Access to improved latrine^2^	17.2	16.4	14.5	15.7
Always or usually use primary toilet for defecation	94.2	95.8	95.9	95.7
Daily defecating in the open, children aged 0 to <3 y	77.8	75.6	77.1	78.3
Human feces observed in compound	7.9	8.4	9.0	9.2
Handwashing, %				
Has water ≤2 m of handwashing location	24.1	27.5	28.4	28.1
Has soap ≤2 m of handwashing location	8.6	13.8	10.6	10.5
Food security, %				
Prevalence of moderate to severe household hunger^3^	11.6	10.0	13.1	11.1

1 Values are means ± SDs except where noted. Enrollment characteristics excluding known pregnancy loss are presented. N, nutrition; WASH, water, sanitation, and hygiene; WSH, water, sanitation, and handwashing.

2 Drinking water and sanitation facilities were considered “improved” if they met the WHO/UNICEF Joint Monitoring Program criteria. Improved drinking water was defined as piped water, public tap, tube well or borehole, protected well, or protected spring. Improved sanitation was defined as flush/pour flush pit latrine, ventilated improved pit latrine, pit latrine with slab, or composting toilet.

3 Moderate to severe hunger defined using the Household Hunger Scale (21).

**FIGURE 2:**
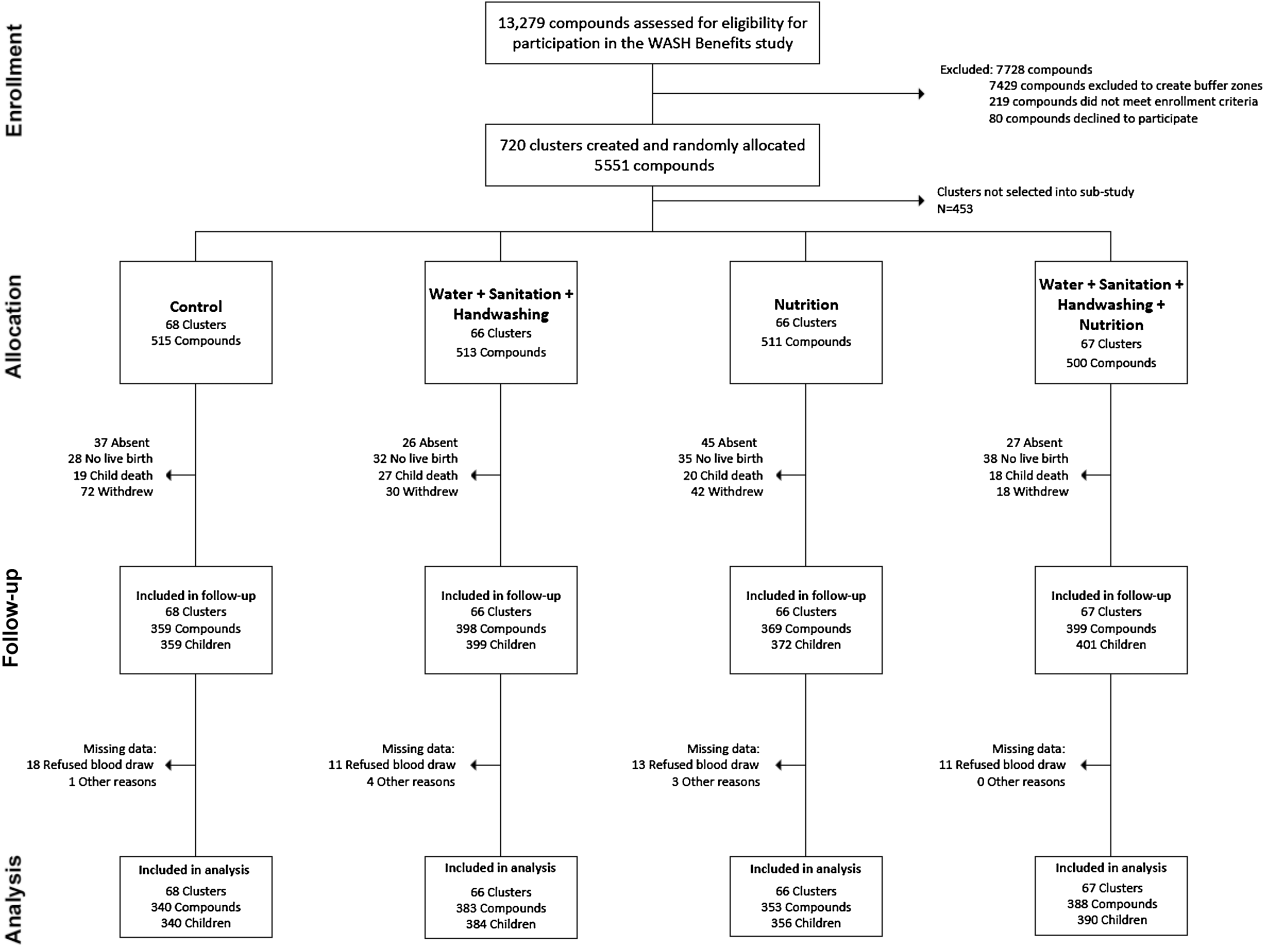
Summary of participant enrollment, random assignment, retention, and analysis in the WASH Benefits Bangladesh Study. WASH, water, sanitation, and hygiene.

In the Bangladesh trial, study enrollment occurred between May 2012 and July 2013. A total of 267 clusters with 2039 compounds were included in the substudy, balanced across the 4 intervention arms (**Figure 2**). At the follow-up point, between March 2015 and March 2016, we had data on anemia or micronutrient status for 1470 children. Follow-up rates differed somewhat between groups, with the lowest rates in the control group (70% of liveborn children) compared with the N (74%), WSH (80%), and WSH+N (84%) groups. Baseline characteristics were balanced across groups (**Table 2**). The mean age of mothers was ∼24 y and, on average, they had had 1 previous birth. More of the mothers had completed at least primary education (∼70%) than the fathers (∼60%). Families lived in communal compounds with an average of 10–11 persons/compound. More than 70% of households had access to a tube well as their primary water source, but virtually no households reported treating their drinking water. Slightly more than half owned their latrine. A low proportion of households had a dedicated handwashing station near their latrine or kitchen, and soap was uncommon. Food insecurity affected ∼30% of households. Individuals who were lost to follow-up or who had missing hemoglobin outcome data were similar in terms of most characteristics to those included in the substudy (**Supplemental Table 4**). Included participants also had similar baseline characteristics to those not selected into the substudy. Just as in Kenya, the adherence to LNS supplementation was high (>90% of expected sachets consumed) at both time points of measurement among the age-eligible children (**Supplemental Table 5**).

**TABLE 2:** Enrollment characteristics by intervention group in the WASH Benefits Bangladesh Trial^1^

	Group			
	Active control	WSH	N	WSH+N
Compounds, *n*	487	481	476	462
Maternal				
Age, y	23.4 ± 4.9	24.3 ± 5.4	23.7 ± 5.0	24.1 ± 5.4
Height, cm	150.8 ± 5.2	150.5 ± 5.5	150.2 ± 5.5	150.0 ± 5.2
Previous births, *n*	1.2 ± 1.3	1.4 ± 1.4	1.4 ± 1.6	1.5 ± 1.5
Completed at least primary education, %	77.8	73.4	71.2	68.0
Paternal, %				
Completed at least primary education	63.0	60.3	57.8	57.1
Works in agriculture	24.0	28.5	33.4	28.4
Household				
People per compound, *n*	10.0 ± 5.8	11.2 ± 6.3	11.5 ± 6.5	11.2 ± 6.7
People per household, *n*	4.7 ± 2.5	4.6 ± 2.0	4.7 ± 2.3	4.9 ± 2.2
Children aged <18 y in the household, *n*	1.5 ± 1.3	1.6 ± 1.2	1.6 ± 1.3	1.7 ± 1.3
Has electricity, %	58.9	60.9	60.5	60.8
Has a cement floor, %	16.4	12.1	11.8	11.7
Has an iron roof, %	99.4	98.3	98.3	98.5
Drinking water, %				
Shallow tube well primary water source	72.3	75.3	70.6	70.3
Stored water observed at home	52.2	45.1	48.1	51.5
Reported treating currently stored water	0.2	0	0	0.2
Sanitation, %				
Own any latrine	59.8	54.1	54.0	51.5
Open defecation by adult	5.1	8.3	8.8	8.4
Open defecation by child aged <8 y	8.0	9.1	9.2	9.7
Human feces observed in house or child play area	5.5	7.7	9.2	8.0
Handwashing, %				
Within 6 steps of latrine				
Has water	17.5	11.2	9.5	11.9
Has soap	9.4	6.9	5.5	6.1
Within 6 steps of kitchen				
Has water	10.3	9.1	9.5	9.1
Has soap	3.7	2.3	4.0	3.0
Food security, %				
Prevalence of food insecurity^2^	27.7	33.1	30.3	29.1

1 Values are means ± SDs except where noted. Characteristics excluding known pregnancy loss are presented. N, nutrition; WASH, water, sanitation, and hygiene; WSH, water, sanitation, and handwashing.

2 Any level of food insecurity assessed using the Household Food Insecurity Access Scale (20).

### Kenya results

**FIGURE 3:**
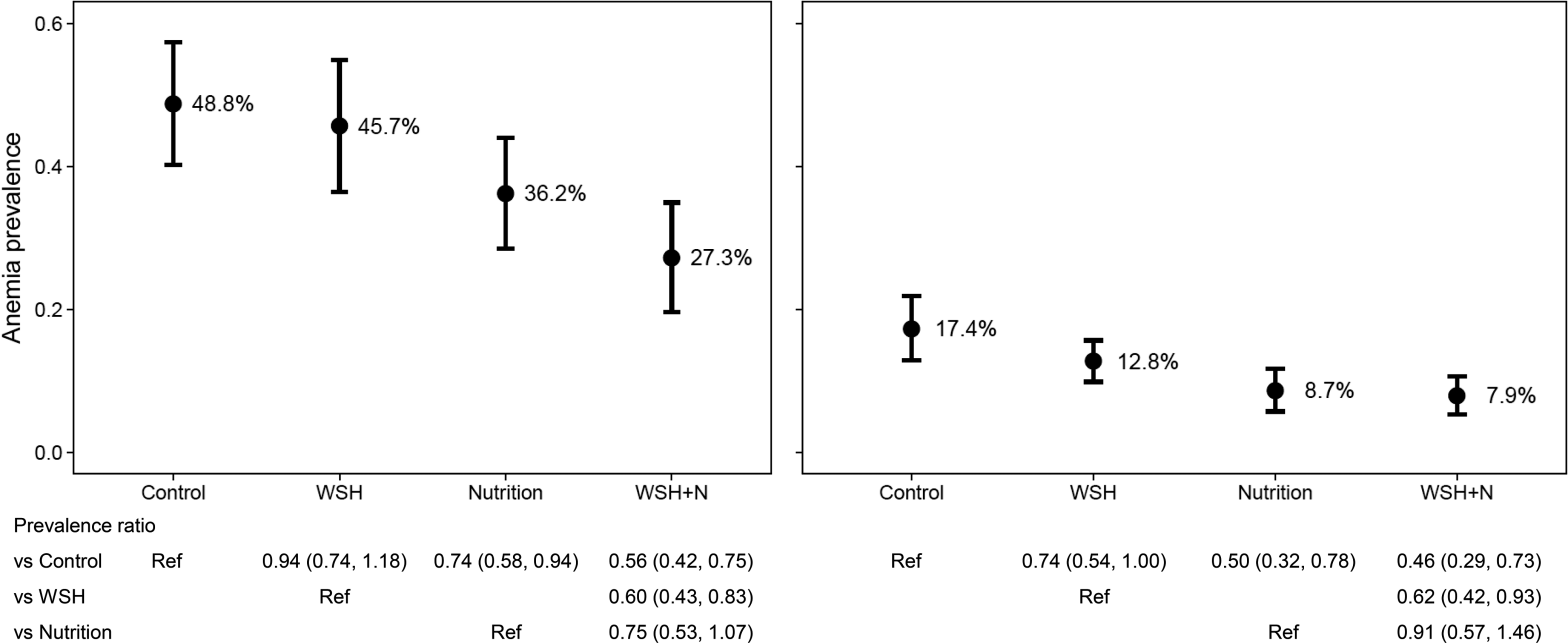
Prevalence (95% CI) of anemia in each intervention group in the WASH Benefits Kenya (A) and Bangladesh (B) Trials. Prevalence ratios and 95% CIs were derived from generalized linear models using a binomial distribution and log link with robust SEs controlling for clustering at the block level. N, nutrition; Ref, reference; WASH, water, sanitation, and hygiene; WSH, water, sanitation, and handwashing.

In the control group, the mean hemoglobin concentration was 110 g/L and 48.8% were anemic at the 2-y follow-up point, when children had a mean ± SD age of 22.1 ± 1.8 mo (**Table 3**, **Figure 3**). The prevalences of mild, moderate, and severe anemia were 29%, 19%, and 0.6%, respectively. Median iron status biomarker concentrations were 18.0 µg/L for ferritin, 12.1 mg/L for sTfR, and 6.0 ng/mL for hepcidin (**Table 4**). The estimated prevalence of iron deficiency differed greatly depending on which biomarker was used in the definition. Using only the ferritin cutoff of <12 µg/L, the prevalence of iron deficiency was 29.9%, whereas using only the sTfR cutoff of >8.3 mg/L, the prevalence was 79.6%. The prevalence of low hepcidin (<5.5 ng/mL) was 48.1%. Using either low ferritin or high sTfR, the estimated prevalence of iron deficiency was 80.9% and the prevalence of iron deficiency anemia was 40.8%. The prevalence of low vitamin B-12 (<221 pmol/L) was 21.8% and folate deficiency was 9.6%.

**TABLE 3:** Comparison of mean differences in hemoglobin between intervention groups in the WASH Benefits Kenya and Bangladesh Trials^1^

	*n*	Mean ± SD hemoglobin, g/L	Mean difference vs. control (95% CI)	Mean difference vs. WSH (95% CI)	Mean difference vs. N (95% CI)
Kenya					
Control	162	110 ± 13.1	Ref	—	—
WSH	138	110 ± 13.1	−0.43 (−2.79, 1.94)	Ref	—
N	196	113 ± 12.7	3.20 (0.41, 6.00)*	—	Ref
WSH+N	154	114 ± 12.2	4.44 (1.36, 7.51)**	4.86 (2.20, 7.53)***	1.23 (−1.82, 4.28)
Bangladesh					
Control	340	119 ± 9.7	Ref	—	—
WSH	384	119 ± 9.2	0.50 (−0.98, 1.97)	Ref	—
N	356	121 ± 8.2	2.68 (1.11, 4.25)***	—	Ref
WSH+N	390	121 ± 8.4	2.44 (0.91, 3.96)**	1.94 (0.74, 3.14)**	−0.25 (−1.54, 1.05)

1 Mean differences and 95% CIs were derived from generalized linear models with robust SEs controlling for clustering at the block level. *P < 0.05, **P < 0.01, ***P < 0.001. N, nutrition; Ref, reference; WASH, water, sanitation, and hygiene; WSH, water, sanitation, and handwashing.

**TABLE 4:** Comparison of biomarkers of micronutrient status between intervention groups in the WASH Benefits Kenya Trial^1^

Outcome	*n*	Median (Q1, Q3)	Percentage difference vs. control (95% CI)	Percentage difference vs. WSH (95% CI)	Percentage difference vs. N (95% CI)
RBP, µmol/L					
Control	157	0.81 (0.65, 1.03)	Ref	—	—
WSH	125	0.86 (0.69, 1.04)	5.6 (−2.5, 14.5)	Ref	—
N	173	0.91 (0.77, 1.09)	15.8 (6.9, 25.5)***	—	Ref
WSH+N	144	0.93 (0.78, 1.08)	14.8 (6.7, 23.6)***	8.7 (0.7, 17.3)*	−0.9 (−7.6, 6.4)
Ferritin, µg/L					
Control	157	18.0 (10.0, 30.8)	Ref	—	—
WSH	125	15.6 (9.0, 30.2)	−8.3 (−25.9, 13.6)	Ref	—
N	173	31.9 (19.8, 55.8)	70.1 (41.9, 103.9)***	—	Ref
WSH+N	144	32.1 (20.4, 49.0)	66.0 (35.6, 103.1)***	80.9 (48.1, 121.1)***	−2.4 (−18.0, 16.1)
sTfR, mg/L					
Control	157	12.1 (8.8, 16.9)	Ref	—	—
WSH	125	12.0 (8.3, 18.0)	−1.1 (−10.1, 8.9)	Ref	—
N	173	8.5 (7.3, 12.4)	−23.2 (−29.9, −15.8)***	—	Ref
WSH+N	144	9.6 (7.3, 12.8)	−20.2 (−28.5, −10.8)***	−19.3 (−27.0, −10.9)***	3.9 (−4.3, 12.9)
Hepcidin, ng/mL					
Control	158	6.0 (2.3, 13.5)	Ref	—	—
WSH	131	5.1 (1.6, 10.6)	−22.6 (−46.7, 12.5)	Ref	—
N	182	13.0 (6.8, 22.2)	123.4 (56.8, 218.1)***	—	Ref
WSH+N	149	14.1 (7.0, 22.4)	109.5 (46.3, 200.1)***	170.6 (80.7, 305.2)***	−6.2 (−27.9, 22.0)
Vitamin B-12, pmol/L					
Control	156	301.0 (225.5, 426.7)	Ref	—	—
WSH	126	303.8 (231.9, 407.9)	−4.2 (−14.6, 7.4)	Ref	—
N	178	357.9 (269.5, 519.2)	18.5 (7.3, 30.9)***	—	Ref
WSH+N	142	371.6 (283.0, 515.7)	20.9 (7.3, 36.2)**	26.3 (12.8, 41.4)***	2.0 (−9.7, 15.2)

1 Percentage differences and 95% CIs were derived from generalized linear models with robust SEs controlling for clustering at the block level. Outcomes were log-transformed for analysis. *P < 0.05, **P < 0.01, ***P < 0.001. N, nutrition; RBP, retinol-binding protein; Q, quartile; Ref, reference; sTfR, soluble transferrin receptor; WASH, water, sanitation, and hygiene; WSH, water, sanitation, and handwashing.

The 2 N intervention groups (N and WSH+N) had higher concentrations of hemoglobin, RBP, ferritin, hepcidin, and vitamin B-12 and lower sTfR (Tables3 and 4). There were no significant differences between the WSH+N and N groups for any of these outcomes.

Compared with the control group, there was a 26% and 44% lower prevalence of anemia in the N and WSH+N groups, respectively (Figure 3). This corresponded to a 12.5 and 21.5 percentage point lower anemia prevalence in those 2 groups, respectively (**Supplemental Table 6**). There was a 9.0 percentage point lower prevalence of anemia in the WSH+N group compared with the N group, but this difference was not significant (*P* = 0.102). There were significantly lower prevalences of iron deficiency, iron deficiency anemia, vitamin A deficiency, and vitamin B-12 depletion or deficiency in both of the groups that received the N intervention package (****Table 5****). Folate deficiency was nearly eliminated in those 2 groups, and there was a concurrent higher proportion of children with high folate concentrations.

There were no differences in mean hemoglobin or micronutrient status markers between the WSH and the control groups (Tables3 and 4). Similarly, there were no differences in the prevalence of anemia or any of the micronutrient deficiencies between the WSH and the control groups (Table 5).

**TABLE 5:** Prevalence ratios between groups in the WASH Benefits Kenya Trial^1^

Outcome	*n*	Prevalence, %	Ratio vs. control (95% CI)	Ratio vs. WSH (95% CI)	Ratio vs. N (95% CI)
Low hepcidin (<5.5 ng/mL)					
Control	158	48.1	Ref	—	—
WSH	131	50.4	1.05 (0.85, 1.30)	Ref	—
N	182	17.0	0.35 (0.25, 0.51)***	—	Ref
WSH+N	149	23.5	0.49 (0.33, 0.71)***	0.47 (0.32, 0.69)***	1.38 (0.82, 2.32)
Low ferritin (<12 µg/L)					
Control	157	29.9	Ref	—	—
WSH	125	37.6	1.26 (0.92, 1.71)	Ref	—
N	173	13.9	0.46 (0.29, 0.74)**	—	Ref
WSH + N	144	9.0	0.30 (0.17, 0.55)***	0.24 (0.13, 0.43)***	0.65 (0.34, 1.24)
High sTfR (>8.3 mg/L)					
Control	157	79.6	Ref	—	—
WSH	125	75.2	0.94 (0.82, 1.08)	Ref	—
N	173	54.3	0.68 (0.60, 0.78)***	—	Ref
WSH+N	144	61.1	0.77 (0.65, 0.91)**	0.81 (0.69, 0.94)**	1.12 (0.94, 1.35)
Iron deficiency (ferritin < 12 µg/L or sTfR > 8.3 mg/L)					
Control	157	80.9	Ref	—	—
WSH	125	77.6	0.96 (0.84, 1.10)	Ref	—
N	173	57.2	0.71 (0.62, 0.80)***	—	Ref
WSH + N	144	62.5	0.77 (0.66, 0.90)**	0.81 (0.69, 0.94)**	1.09 (0.92, 1.29)
Iron deficiency anemia (anemic and iron deficient)					
Control	152	40.8	Ref	—	—
WSH	131	38.2	0.94 (0.73, 1.20)	Ref	—
N	189	23.8	0.58 (0.43, 0.78)***	—	Ref
WSH+N	154	20.1	0.49 (0.33, 0.73)***	0.53 (0.35, 0.79)**	0.85 (0.54, 1.33)
Vitamin A deficiency (RBP *<*0.83 µmol/L)					
Control	157	52.9	Ref	—	—
WSH	125	44.0	0.83 (0.63, 1.10)	Ref	—
N	173	34.7	0.66 (0.50, 0.87)**	—	Ref.
WSH+N	144	28.5	0.54 (0.40, 0.72)***	0.65 (0.45, 0.93)*	0.82 (0.59, 1.15
Vitamin B-12 deficiency (*<*150 pmol/L)					
Control	156	4.5	Ref	—	—
WSH	126	5.6	1.24 (0.36, 4.20)	Ref	—
N	178	3.9	0.88 (0.41, 1.90)	—	Ref
WSH+N	142	2.8	0.63 (0.20, 2.01)	0.51 (0.13, 1.96)	0.72 (0.24, 2.13)
Vitamin B-12 depletion or deficiency (*<*221 pmol/L)					
Control	156	21.8	Ref	—	—
WSH	126	21.4	0.98 (0.63, 1.52)	Ref	—
N	178	12.9	0.59 (0.39, 0.90)*	—	Ref
WSH+N	142	9.9	0.45 (0.25, 0.83)*	0.46 (0.25, 0.86)*	0.76 (0.42, 1.37)
Folate deficiency (*<*10 nmol/L)					
Control	156	9.6	Ref	—	—
WSH	129	14.0	1.45 (0.69, 3.06)	Ref	—
N	178	1.1	0.12 (0.03, 0.54)**	—	Ref
WSH+N	142	0.7	0.07 (0.01, 0.49)**	0.05 (0.01, 0.38)**	0.63 (0.06, 6.98)
High folate (*>*45.3 nmol/L)					
Control	156	3.2	Ref	—	—
WSH	129	7.8	2.42 (0.83, 7.08)	Ref	—
N	178	42.1	13.15 (5.97, 28.94)***	—	Ref
WSH+N	142	35.9	11.21 (4.79, 26.21)***	4.63 (2.53, 8.49)***	0.85 (0.63, 1.16)

1 Prevalence ratios and 95% CIs were derived from generalized linear models using a binomial distribution and log link with robust SEs controlling for clustering at the block level. *P < 0.05, **P < 0.01, ***P < 0.001. N, nutrition; RBP, retinol-binding protein; Ref, reference; sTfR, soluble transferrin receptor; WASH, water, sanitation, and hygiene; WSH, water, sanitation, and handwashing.

In the control group, the prevalence of malaria was 18.9% and inflammation as measured by CRP or AGP was present in 53.5%; these did not differ between study arms (**Supplemental Table 7**). Correction for inflammation reduced the estimated prevalence of vitamin A deficiency but slightly increased the prevalence of iron deficiency and iron deficiency anemia across all groups. Estimated differences between groups were similar to those calculated using the non–inflammation-corrected values.

The results were similar after adjustment for covariates and in the IPCW sensitivity analyses (**Supplemental Table 8**). There was no evidence of effect modification by age, sex, or household hunger score (*P* > 0.1 for all; data not shown), although a small sample size of households with moderate to severe hunger limited our ability to detect a potential interaction. There was some evidence of effect modification with hemoglobinopathy trait, but there was not a consistent pattern in the stratified analysis (**Supplemental Table 9**).

### Bangladesh results

At follow-up, children had a mean ± SD age of 28.0 ± 1.9 mo. In the control group, the mean hemoglobin concentration was 119 g/L (Table 3) and the prevalence of anemia was 17.4% (Figure 3). The prevalences of mild, moderate, and severe anemia were 15.4%, 1.8%, and 0%, respectively. Approximately one-third (34.8%) of children suffered from iron deficiency, defined as either low ferritin or high sTfR, and 9.7% had iron deficiency anemia. Vitamin A deficiency and low vitamin B-12 affected 16.1% and 20.7% of children, respectively, whereas folate deficiency was rare (<3%). Only 8.7% of children had elevated CRP concentrations).

In the 2 N intervention groups (N and WSH+N), hemoglobin, ferritin, hepcidin, and vitamin B-12 concentrations were significantly higher, whereas sTfR was significantly lower compared with the control group (Table3 and **Table 6**). The WSH group did not differ from the control group in any of these biomarkers. RBP concentrations did not differ between groups. Folate concentrations were significantly lower in the WSH and WSH+N groups. There were no significant differences between the WSH+N group and the N group.

**TABLE 6:** Comparison of biomarkers of micronutrient status between intervention groups in the WASH Benefits Bangladesh Trial^1^

Outcome	*n*	Median (Q1, Q3)	Percentage difference vs. control (95% CI)	Percentage difference vs. WSH (95% CI)	Percentage difference vs. N (95% CI)
RBP, µmol/L					
Control	310	1.08 (0.91, 1.28)	Ref	—	—
WSH	370	1.05 (0.86, 1.25)	−3.1 (−8.4, 2.5)	Ref	—
N	336	1.09 (0.90, 1.28)	0.1 (−5.3, 5.9)	—	Ref
WSH+N	372	1.12 (0.94, 1.29)	2.2 (−2.7, 7.4)	5.5 (1.6, 9.5)**	2.1 (−2.0, 6.3)
Folate, nmol/L					
Control	304	30.2 (22.5, 37.5)	Ref	—	—
WSH	359	26.0 (18.2, 34.0)	−13.9 (−20.6, −6.5)***	Ref	—
N	329	27.8 (19.7, 35.6)	−7.5 (−15.5, 1.2)	—	Ref
WSH+N	363	26.8 (19.6, 35.4)	−8.7 (−15.4, −1.4)*	6.0 (−1.4, 14.0)	−1.2 (−7.4, 5.4)
Ferritin, µg/L					
Control	310	24.2 (14.1, 37.5)	Ref	—	—
WSH	370	25.5 (14.6, 40.7)	5.9 (−5.5, 18.7)	Ref	—
N	336	37.9 (25.8, 51.9)	56.2 (40.9, 73.2)***	—	Ref
WSH+N	372	39.3 (26.4, 53.6)	63.6 (45.8, 83.5)***	54.4 (40.2, 70.2)***	4.7 (−4.6, 15.0)
sTfR, mg/L					
Control	310	7.2 (6.1, 8.5)	Ref	—	—
WSH	370	7.1 (6.1, 8.4)	−1.5 (−7.7, 5.1)	Ref	—
N	336	6.5 (5.9, 7.5)	−12.4 (−17.9, −6.5)***	—	Ref
WSH+N	372	6.7 (6.0, 7.6)	−11.1 (−16.1, −5.7)***	−9.7 (−13.0, −6.4)***	1.5 (−2.7, 5.9)
Hepcidin, ng/mL					
Control	156	13.6 (5.9, 24.3)	Ref	—	—
WSH	217	12.7 (6.3, 26.0)	−5.7 (−29.3, 25.9)	Ref	—
N	178	16.9 (9.7, 28.0)	48.8 (15.7, 91.6)**	—	Ref
WSH+N	181	19.1 (11.8, 31.5)	64.4 (30.6, 107.1)***	74.3 (35.6, 124.0)***	10.5 (−7.2, 31.6)
Vitamin B-12, pmol/L					
Control	304	296.6 (239.8, 394.4)	Ref	—	—
WSH	360	314.5 (250.4, 426.4)	5.1 (−2.1, 12.7)	Ref	—
N	332	348.5 (276.0, 437.4)	11.6 (3.8, 20.0)**	—	Ref
WSH+N	363	344.3 (250.8, 447.3)	11.8 (4.5, 19.7)**	6.5 (0.8, 12.4)*	0.2 (−6.1, 7.0)

1 Percentage differences and 95% CIs were derived from generalized linear models with robust SEs controlling for clustering at the block level. Outcomes were log-transformed for analysis. *P *<* 0.05, **P *<* 0.01, ***P *<* 0.001. N, nutrition; Q, quartile; RBP, retinol-binding protein; Ref, reference; sTfR, soluble transferrin receptor; WASH, water, sanitation, and hygiene; WSH, water, sanitation, and handwashing.

**TABLE 7:** Prevalence ratios between groups in the WASH Benefits Bangladesh Trial^1^

Outcome	*n*	Prevalence, %	Ratio vs. control (95% CI)	Ratio vs. WSH (95% CI)	Ratio vs. N (95% CI)
Low hepcidin (<5.5 ng/mL)					
Control	156	23.7	Ref	—	—
WSH	217	20.7	0.87 (0.59, 1.29)	Ref	—
N	178	9.6	0.40 (0.23, 0.70)**	—	Ref
WSH+N	181	6.6	0.28 (0.16, 0.49)***	0.32 (0.18, 0.58)***	0.69 (0.35, 1.38)
Low ferritin (<12 µg/L)					
Control	310	20.0	Ref	—	—
WSH	370	15.4	0.77 (0.56, 1.05)	Ref	—
N	336	5.4	0.27 (0.16, 0.46)***	—	Ref
WSH+N	372	3.0	0.15 (0.09, 0.25)***	0.19 (0.11, 0.34)***	0.55 (0.29, 1.06)
High sTfR (>8.3 mg/L)					
Control	310	27.1	Ref	—	—
WSH	370	26.8	0.99 (0.75, 1.30)	Ref	—
N	336	12.5	0.46 (0.31, 0.68)***	—	Ref
WSH+N	372	13.2	0.49 (0.33, 0.71)***	0.49 (0.35, 0.69)***	1.05 (0.66, 1.67)
Iron deficiency (ferritin < 12 µg/L or sTfR >8.3 mg/L)					
Control	310	34.8	Ref	—	—
WSH	370	32.2	0.92 (0.73, 1.16)	Ref	—
N	336	15.8	0.45 (0.33, 0.62)***	—	Ref
WSH+N	372	14.8	0.42 (0.30, 0.60)***	0.46 (0.33, 0.63)***	0.94 (0.64, 1.38)
Iron deficiency anemia (anemic and iron deficient)					
Control	329	9.7	Ref	—	—
WSH	378	6.6	0.68 (0.39, 1.18)	Ref	—
N	351	2.6	0.26 (0.11, 0.62)**	—	Ref
WSH+N	387	1.3	0.13 (0.05, 0.35)***	0.20 (0.08, 0.49)***	0.50 (0.16, 1.57)
Vitamin A deficiency (RBP *<*0.83 µmol/L)					
Control	310	16.1	Ref	—	—
WSH	370	19.7	1.22 (0.83, 1.80)	Ref	—
N	336	16.7	1.03 (0.66, 1.62)	—	Ref
WSH+N	372	11.8	0.73 (0.49, 1.11)	0.60 (0.41, 0.87)**	0.71 (0.48, 1.04)
Vitamin B-12 deficiency (*<*150 pmol/L)					
Control	304	2.3	Ref	—	—
WSH	360	3.1	1.33 (0.48, 3.67)	Ref	—
N	332	3.0	1.31 (0.40, 4.26)	—	Ref
WSH+N	363	1.4	0.60 (0.17, 2.08)	0.45 (0.15, 1.31)	0.46 (0.14, 1.51)
Vitamin B-12 depletion or deficiency (*<*221 pmol/L)					
Control	304	20.7	Ref	—	—
WSH	360	16.1	0.78 (0.55, 1.10)	Ref	—
N	332	11.4	0.55 (0.34, 0.89)*	—	Ref
WSH+N	363	16.3	0.78 (0.57, 1.08)	1.01 (0.72, 1.42)	1.42 (0.90, 2.25)
Folate deficiency (*<*10 nmol/L)					
Control	304	2.3	Ref	—	—
WSH	359	5.8	2.54 (1.15, 5.60)*	Ref	—
N	329	2.7	1.19 (0.46, 3.06)	—	Ref
WSH+N	363	2.8	1.20 (0.51, 2.83)	0.47 (0.24, 0.94)*	1.01 (0.40, 2.55)
High folate (*>*45.3 nmol/L)					
Control	304	9.2	Ref	—	—
WSH	359	8.1	0.88 (0.48, 1.60)	Ref	—
N	329	8.5	0.92 (0.48, 1.77)	—	Ref
WSH+N	363	7.4	0.81 (0.45, 1.44)	0.92 (0.58, 1.45)	0.87 (0.52, 1.46)

1 Prevalence ratios and 95% CIs were derived from generalized linear models using a binomial distribution and log link with robust SEs controlling for clustering at the block level. *P < 0.05, **P < 0.01, ***P < 0.001. N, nutrition; RBP, retinol-binding protein; Ref, reference; sTfR, soluble transferrin receptor; WASH, water, sanitation, and hygiene; WSH, water, sanitation, and handwashing.

Similar to the results seen in the continuous concentration measures, the prevalences of anemia and iron deficiency were ∼50% lower in the 2 N groups compared with the control group (Figure 3). For anemia, this corresponded to an ∼9 percentage point reduction in prevalence (**Supplemental Table 10**). Iron deficiency anemia was 74–87% lower and the prevalence almost eliminated (<3%) in these 2 groups. The prevalence of anemia was 26% lower in the WSH group compared with the control, but there was no added benefit of WSH+N over N alone. The prevalence of vitamin A deficiency did not differ between groups (**Table 7**). The prevalence of vitamin B-12 depletion or deficiency was significantly lower by 45% in the N group, but the magnitude of the reduction was attenuated and not statistically significant in the WSH+N group compared with the control group. There was a higher prevalence of folate deficiency in the WSH group compared with the control group and no difference in the prevalence of high folate between any of the groups.

There was a strong seasonal pattern in mean folate concentrations and the timing of sample collection varied between groups (**Supplemental Figure 2**). The other nutrient biomarkers did not show such a strong seasonal pattern. In models adjusted for covariates, including month of sample collection, mean folate concentrations did not differ between groups (**Supplemental Table 11**). All other adjusted group comparisons were similar to the unadjusted estimates.

Correction for inflammation reduced the estimated prevalence of vitamin A deficiency, but slightly increased the prevalence of iron deficiency and iron deficiency anemia across all groups. Estimated differences between groups were similar to those calculated using the non–inflammation-corrected values (**Supplemental Table 12**). The results were also similar in the IPCW analyses (Supplemental Table 11). There was some evidence of effect modification by child age, with an effect of substantially larger magnitude evident on the iron biomarkers in children aged ≤28 mo, the median child age (**Supplemental Table 13**). Specifically, there was a 75–86% higher median ferritin and 20–21% lower median sTfR in the N and WSH+N groups, compared with the control, among children aged ≤28 mo. In children aged >28 mo, there was a more modest 47–53% higher median ferritin and 5–7% lower median sTfR in those 2 groups compared with controls. There was also a significant interaction with food insecurity with regard to vitamin B-12 status: effects were apparent only among children in food-insecure households in the WSH, N, and WSH+N groups compared with controls and no effects were evident in children from food-secure households (**Supplemental Table 14**). There were no significant interactions with child sex or thalassemia trait (*P* > 0.1 for all interaction tests; data not shown).

## Discussion

We found that a nutrition intervention focused on improving IYCF practices together with distribution of LNSs resulted in a significantly and substantially lower prevalence of anemia and iron deficiency in both Kenya and Bangladesh, despite very different control group prevalences of these conditions (48.8% in Kenya and 17.4% in Bangladesh). Iron deficiency anemia was nearly eliminated in the 2 N intervention groups in Bangladesh. There were also reductions in the prevalence of low vitamin B-12 status in both trials. The prevalences of vitamin A deficiency and folate deficiency were reduced in the N arms in Kenya, but not in Bangladesh. In Bangladesh, children in the WSH intervention group had a 26% lower prevalence of anemia compared with the control group, but there were no effects of the WSH intervention on any of the micronutrient biomarkers, and there was no added benefit of combined WSH+N as compared with the individual nutrition-specific intervention.In contrast, in Kenya, there was a 9 percentage point reduction in the prevalence of anemia in the combined WSH+N group compared with N alone, although this difference was not significant.

Although these 2 trials are comparable in many ways, there are a number of notable differences in the design, implementation, and context. The prevalences of anemia and iron deficiency were substantially higher in Kenya than in Bangladesh and so the potential of benefit was greater in Kenya. The prevalence of anemia of 17.4% in Bangladesh was lower than expected. By contrast, the reported prevalence of anemia among preschool children in the 2013 Bangladesh National Micronutrient Status Survey was 33% (44). The difference between our study and the national survey may be partially explained by the variations in sampling methods, because the survey used capillary blood, whereas our study used venous blood sampling. Hemoglobin concentrations are affected by the sampling techniques (45). It is also possible that groundwater iron concentrations in Bangladesh may have explained the lower than expected prevalence of iron deficiency and anemia. Although we selected a study area that had a low groundwater iron concentration (16), the measured iron concentrations were variable (17). The median concentration was 0.91 mg/L (IQR: 0.36, 2.01 mg/L) and 18% of tested tube wells had iron concentrations >3 mg/L (17). One study conducted in an area with a higher median concentration of 7.6 mg/L reported strong associations between groundwater iron concentrations and iron status among women (46).

In the Kenya study, the N intervention package also significantly reduced the prevalences of vitamin A deficiency and folate deficiency compared with controls, whereas this was not apparent in the Bangladesh study. One possible explanation for the variation in estimated impacts on micronutrient status is that there were delays in the timing of sample collection in the Bangladesh study due to security concerns arising from civil unrest, such that children were, on average, 4 mo beyond the age at which they stopped receiving LNSs. Indeed, effect sizes were larger among young children in stratified analyses in that trial. Serum folate is reflective of recent intake (32), so we might not expect this biomarker to be affected 4 mo after supplementation had ceased. Serum ferritin, transferrin receptor, vitamin B-12, and RBP reflect somewhat longer-term status (47, 48). However, effects of these supplements may have begun to wash out after this length of time. In studies of high-dose vitamin A supplementation, serum retinol concentrations reverted to baseline values within ∼4 mo after the point of supplementation (49). However, in addition to supplementation, our trial included behavioral recommendations to increase dietary diversity. We found significant differences in reported dietary diversity in the 2 N groups in Bangladesh (19) but not in Kenya (15). However, even in the Bangladesh control group at the 2-y follow-up, 78% of children met the minimum dietary diversity indicator of ≥4 food groups/d, whereas 92% of children in the N and WSH+N groups met this criterion. Thus, the reported differences in dietary practices between groups may not have been large enough to be reflected in differences in vitamin A or folate status after LNS supplementation ended.

There were somewhat contrasting results on the effects of the WSH intervention on anemia in the 2 trials. In Bangladesh, there was an effect of WSH alone, whereas in Kenya the prevalence of anemia was 9 percentage points lower in the WSH+N group when compared with the nutrition-specific intervention group (N). Although this was not significant in Kenya, the difference was of a large magnitude and suggests that there may be additional benefit from combining WSH with a nutrition intervention package. The lower than expected sample size due to a high rate of missing data reduced the statistical power to detect this difference. Using our empirical data of 36.2% and 27.3% prevalences, an intracluster correlation of 0.108, 41 clusters per arm, and 4 children per cluster, the study would have needed 569 children per group to have 80% power. By contrast, the study included only 205 children in the N group and 167 children in the WSH+N group.

In the Kenya trial, the uptake of the WSH interventions was lower than in Bangladesh and there was no effect on diarrhea (9, 10). A recent systematic review of observational studies reported that poor sanitation (unimproved or open defecation) was strongly associated with anemia (2); however, the biological pathway is unclear. The primary hypothesized mechanisms through which WASH interventions may affect anemia risk would be a reduction in soil-transmitted helminths, diarrhea, or inflammation. Among soil-transmitted helminths, hookworm and *Trichuris* infections have the strongest associations with anemia risk (4). Adult hookworms feed on the blood of the host and the resulting blood loss can contribute to iron deficiency anemia (50). We found that the prevalence of hookworm infection was 9% in control-group children in Bangladesh, which was significantly lower at ∼6% in the WSH and WSH+N groups (A Ercumen, J Benjamin-Chung, BF Arnold, A Lin, AE Hubbard, CP Stewart, SM Parvez, L Unicomb, ML Rahman, R Haque, et al.; unpublished results, 2018). In Kenya, in contrast, hookworm prevalence was 2% and there was no effect of the interventions on hookworm infection (AJ Pickering, SM Njenga, L Steinbaum, CP Stewart, A Lin, BF Arnold, HN Dentz, M Mureithi, B Chieng, J Swarthout, et al.; unpublished results, 2018). *Trichuris* was detected in ∼7% of children in Bangladesh and ∼1% in Kenya, but the prevalence was not affected by these interventions in either country. The difference between groups in hookworm prevalence may have been too small to explain the difference in anemia between the WSH group and the control group. There was a reduction in the prevalence of diarrhea in Bangladesh, but the magnitude of that difference was <2 percentage points (9). There was no evidence of an effect of the interventions on inflammation using the biomarkers that we measured. There may be other pathways that we have been unable to explore in these data, but future analyses will explore other measures of inflammation, enteropathy, and morbidity over the multiple time points when these data were collected during the trial. Given that the upper bound of the 95% CI of the effect estimates was at or just above 1.0, it is also possible that the observed effects in the 2 trials were due to chance.

This study adds to the literature on the effects of IYCF interventions on reducing anemia and iron deficiency. Multiple micronutrient powders have a strong evidence base and they are currently recommended by the WHO (7). More recently, investigators have begun to examine the effects of LNSs on anemia and micronutrient status. In a trial in Burkina Faso, provision of LNSs plus malaria and diarrheal disease treatment led to a lower prevalence of anemia and iron deficiency and elevated RBP concentrations (51). It is impossible to disentangle the effects of LNSs from the effects of the disease control intervention in that trial, however. In Honduras, an LNS intervention reduced vitamin A and B-12 deficiencies, but did not affect iron, zinc, or riboflavin status, although adherence to the intervention protocol was low (52). Dietary diversification strategies may also improve micronutrient status and reduce the risk of anemia. Increased consumption of animal-source foods, which contain highly bioavailable sources of iron, vitamin B-12, vitamin A, and other micronutrients, has been shown to reduce the risk of anemia in young children (53, 54).

In Kenya, folate deficiency was nearly eliminated by the intervention, but there was a substantially higher prevalence of high folate concentrations (>45.3 nmol/L). Some concern has been raised about folic acid supplementation or fortification in high-malaria-burden settings because of a reduction in the therapeutic efficacy of sulfadoxine-pyrimethamine drugs (55). Whereas these drugs are often provided to pregnant women as part of the government's intermittent preventive treatment policy, children receive either sulfadoxine-pyrimethamine or artemisinin combination therapy drugs for treatment of malaria (56). Some have also suggested that high blood folate concentrations may exacerbate malaria parasitemia independent of treatment efficacy (57). Although there was not any evidence of a difference in malaria prevalence between groups, we lack data on malaria severity, drug administration, and drug efficacy in this study. Nevertheless, our data suggest that the folic acid dose in the LNS formulation could potentially be reduced because of low prevalences of deficiency in both sites and minimal benefit of supplementation.

The strengths of these studies include their randomized designs and high adherence to the LNS supplementation recommendations. The factorial design enabled us to examine the potential for additive effects of combined N and WSH interventions. The outcome measures were objective and therefore not subject to reporting errors or bias. All primary analyses were prespecified and data analysis was replicated by 2 investigators working with blinded data sets. The studies are limited, however, in a number of important ways. In both trials, we assessed anemia at only 1 time point. Further, the estimated prevalences of iron deficiency and iron deficiency anemia should be viewed with caution because the biomarkers of ferritin and sTfR are known to be affected by inflammation, malaria, and the presence of hemoglobinopathies (40, 41). We have presented our primary analysis uncorrected for inflammation because of the possibility that inflammation could be on the causal pathway, but also presented the inflammation-adjusted results in Supplemental Tables 7 and 12. The correction for inflammation did not substantially change the interpretation of the main results of this analysis. We lack data on birth weight, a factor known to be associated with iron status and anemia (58), as well as biomarkers of other micronutrients associated with anemia, including riboflavin and zinc. In Kenya, there were high rates of refusal for the blood draw, effectively halving the sample size of the baseline cohort when combined with other reasons for losses to follow-up. This high rate of attrition could have led to some selection bias in the study sample. However, losses were generally balanced across groups, and the IPCW analysis results, which reweighted the analysis population to reflect the original enrolled substudy population, were highly consistent with the complete case analysis. Second, there was low adherence to some of the promoted WSH practices during the second year of the trial in Kenya. Objective indicators of handwashing and water treatment were low in the second year of the trial preceding the biological sample collection, but sanitation access was high (10). In Bangladesh, the interpretation is complicated by the delayed timing of sample collection relative to when LNS supplementation ceased and by the seasonal imbalance in sample collection between groups. We were able to statistically control for seasonality, which did affect the estimated effects on folate deficiency.

We conclude that a nutrition intervention providing LNSs with behavior change counseling focused on improving IYCF practices reduced the risk of anemia, iron deficiency, and low vitamin B-12 status in young children. The consistency in findings across both trials suggests that these results can be generalized to other populations in which anemia, iron, and vitamin B-12 deficiencies are common. Impacts on other micronutrient deficiencies were variable between sites, likely due to the timing of sample collection.

## Supplementary Material

nqy239_Supplemental_File

## Notes

Supported by Global Development grant OPPGD759 from the Bill & Melinda Gates Foundation to the University of California, Berkeley (to JMC), with additional funding from Thrasher Research Fund award 11860 (to CPS) and US Agency for International Development (USAID) grant AID-OAA-F-13-00040 to Innovations for Poverty Action (to CN). This article was made possible by the generous support of the American people through USAID.

Supplemental Tables 1–14 and Supplemental Figures 1 and 2 are available from the “Supplementary data” link in the online posting of the article and from the same link in the online table of contents at https://academic.oup.com/ajcn/.

Present address for MK: One Acre Fund, Nairobi, Kenya.

Present address for GR: Waterborne Disease Prevention Branch, Division of Foodborne, Waterborne, and Environmental Diseases, CDC, 1600 Clifton Road, Atlanta, GA 30329.

Present address for CN: Mathmatica Policy Research, 1100 First Street, NE, 12th Floor, Washington, DC 20002.

Present address for AJP: Department of Civil and Environmental Engineering, Tufts University, 200 College Avenue, Medford, MA 02155.

Abbreviations used: AGP, α-1 acid glycoprotein; CHP, community health promoter; CRP, C-reactive protein; IPCW, inverse probability of censoring–weighted; IYCF, infant and young child feeding; KEMRI, Kenya Medical Research Institute; LNS, lipid-based nutrient supplement; N, nutrition; RBP, retinol-binding protein; sTfR, soluble transferrin receptor; WASH, water, sanitation, and hygiene; WSH, water, sanitation, and handwashing.
