# Supplementary material for: Effects of lipid-based nutrient supplements and infant and young child feeding counseling with or without improved water, sanitation, and hygiene (WASH) on anemia and micronutrient status: results from 2 cluster-randomized trials in Kenya and Bangladesh
Source: Am J Clin Nutr. 2019 Jan 9;109(1):148–64. doi: 10.1093/ajcn/nqy239 (PMC6358037; doi:10.1093/ajcn/nqy239)
Supplement: nqy239_Supplemental_File [file nqy239_supplemental_file.pdf]

Effects of lipid based nutrient supplements and infant and young child feeding counseling with or without improved WASH on anemia and micronutrient status: results from two cluster randomized trials in Kenya and Bangladesh

Supplemental Table 1: Nutrient Content of the Lipid-based Nutrient Supplement (LNS) in the WASH Benefits Study compared to the WHO/FAO Recommended Nutrient Intakes (RNI) for children 1-3 years

Supplemental Figure 1: Groundwater iron concentration in Bangladesh and the WASH Benefits subsample study sites.

Supplemental Table 2: Enrollment characteristics by presence in study sample in the WASH Benefits Kenya Trial

Supplemental Table 3: Intervention adherence by group in the WASH Benefits Kenya Trial

Supplemental Table 4: Enrollment characteristics by presence in study sample in the WASH Benefits Bangladesh Trial

Supplemental Table 5: Intervention adherence by group in the WASH Benefits Bangladesh Trial

Supplemental Table 6: Prevalence differences comparing each intervention group to the control in the WASH Benefits Kenya Trial

Supplemental Table 7: Prevalence ratio for iron deficiency, iron deficiency anemia, and vitamin A deficiency after correcting values for inflammation and malaria status, comparing each intervention group to the control in the WASH Benefits Kenya Trial

Supplemental Table 8: Comparison of mean differences between intervention groups after adjusting for covariates and using inverse probability of censoring weighted (IPCW) analysis in the WASH Benefits Kenya Trial

Supplemental Table 9: Effect modification with genetic trait in the WASH Benefits Kenya Trial

Supplemental Table 10: Prevalence differences comparing each intervention group to the control in the WASH Benefits Bangladesh Trial

Supplemental Figure 2: Mean serum folate concentration by intervention group over the study period in the WASH Benefits Bangladesh Trial

Supplemental Table 11: Comparison of mean differences between intervention groups: unadjusted, after adjusting for covariates, and using inverse probability of censoring weighted (IPCW) analysis in the WASH Benefits Bangladesh Trial

Supplemental Table 12: Prevalence ratio for iron deficiency, iron deficiency anemia, and Vitamin A deficiency after correcting values for inflammation, comparing each intervention group to the control in the WASH Benefits Bangladesh Trial

Supplemental Table 13: Effect modification with child age in the WASH Benefits Bangladesh Trial

Supplemental Table 14: Effect modification with food security in the WASH Benefits Bangladesh Trial

References

Supplemental Table 1. Nutrient Content of the Lipid-based Nutrient Supplement (LNS) in the WASH Benefits Study compared to the WHO/FAO Recommended Nutrient Intakes (RNI) for children 1-3 years[1]

Nutrient	Unit	RNI	LNS nutrient content		
			Content	% RNI	Chemical form
Daily Dose	g		20		
Energy	kcal		118		
Fat	g		9.6		
Linoleic acid	g		4.46		
Alpha-linolenic acid	g		0.58		
Protein	g		2.6		
Vitamins					
Vitamin A	µg	400	400	100%	Retyinyl acetate
Vitamin D	µg	5	5	100%	Cholecalciferol (D3)
Vitamin E	mg	5	6	120%	DL-alpha-tocopherol acetate
Vitamin K	µg	15	30	200%	Phylloquinone 5%
Vitamin C	mg	30	30	100%	L-ascorbic acid
Biotin	µg	8	NA		
Folic acid	µg	150	150	100%	Pteroyl monoglutamic acid
Thiamin (B1)	mg	0.5	0.5	100%	Thiamin hydrochloride
Riboflavin (B2)	mg	0.5	0.5	100%	Riboflavin
Niacin	mg	6	6	100%	Niacinamide
Pantothenic acid (B5)	mg	2	2	100%	Calcium pantothenate
Vitamin B6	mg	0.5	0.5	100%	Pyridoxine hydrochloride
Vitamin B12	µg	0.9	0.9	100%	Cyanocobalamin (0.1%)
Minerals					
Calcium	mg	500	280	56%	Tri-calcium phosphate
Copper[2]	mg	0.34	0.34	100%	Encapsulated copper sulfate
Iodine	µg	90	90	100%	Potassium iodate
Iron[3,4]	mg	11.6	9	78%	Encapsulated ferrous sulfate [Bangladesh]<br>Ferrous fumarate [Kenya]
Magnesium	mg	60	40	67%	Magnesium citrate
Manganese	mg	1.2	1.2	100%	Manganese sulfate
Phosphorus	mg	460	190	41%	Tri-calcium phosphate & Di-potassium phosphate
Potassium	mg		200		Di-potassium phosphate & potassium chloride
Selenium	µg	17	20	118%	Sodium selenite 1.5%
Zinc[3]	mg	8.3	8	96%	Zinc sulfate

1. RNI=Recommended Nutrient Intake; LNS=Lipid-based nutrient supplement, produced by Nutriset (Malaunay, France)
2. The Institute of Medicine RDA level for copper for infants 1-3 y is shown here (1).
3. The RNI for iron and zinc is that assumed under a diet of low bioavailability (2).
4. The chemical form of iron varied between the two countries. In Kenya, an interaction between the encapsulated ferrous sulfate and the local millet flour caused discolorations to appear in the food when mixed.

Supplemental Figure 1. Groundwater iron concentration in Bangladesh and the WASH Benefits subsample study sites. Groundwater iron data obtained from the DPHE/BGS National Hydrochemical Survey, 2000.

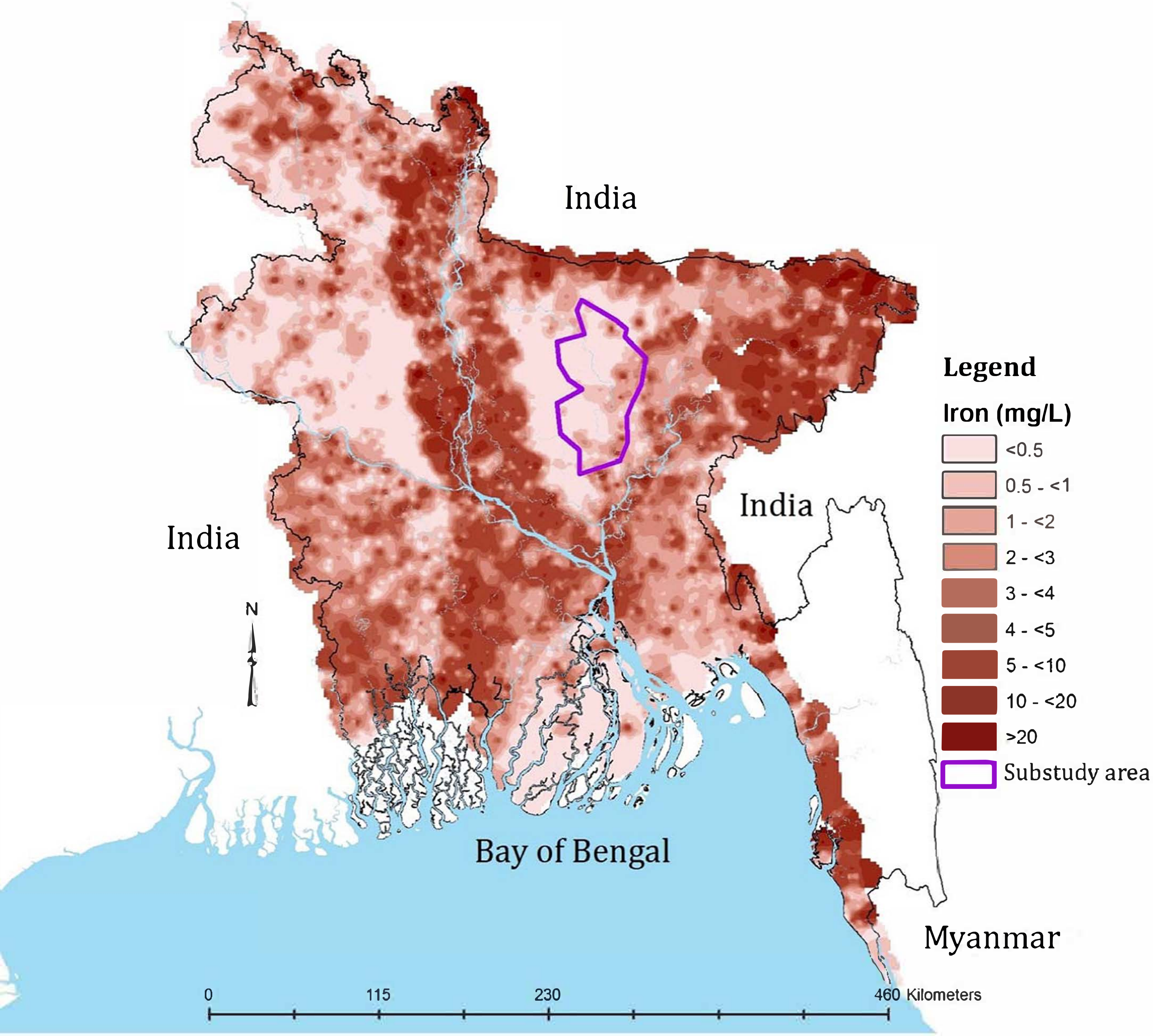

Supplemental Table 2: Enrollment characteristics by presence in study sample in the WASH Benefits Kenya Trial[1]

	Substudy		
	**Included in the analysis**	**Missing data**	**Non-substudy households**
No. of households:	(N=687)	(N=1617)	(N=5942)
**Maternal**			
Age (years)	26.6 ± 6.3	25.4 ± 6.1	26.0 ± 6.4
Maternal height (cm)	160.4 ± 6.1	160.2 ± 5.8	160.2 ± 5.9
Primiparous (%)	19.1	26.5	24.0
Completed at least primary (%)	46.6	48.1	47.4
**Paternal**			
Completed at least primary (%)	63.3	60.1	61.8
Works in agriculture (%)	46.5	44.2	41.7
**Household**			
Number of people per compound	9.0 ± 5.8	8.4 ± 6.1	8.0 ± 5.4
Number of children <18 years in the household	3.1 ± 2.0	2.7 ± 1.9	2.8 ± 2.0
Has electricity (%)	6.3	5.9	7.1
Has a cement floor (%)	4.4	5.7	5.9
Has an iron roof (%)	61.0	62.5	67.7
**Drinking Water**			
Walking time to primary water source (min)	9.9 ± 10.5	10.3 ± 11.1	11.3 ± 13.0
Primary drinking water source is improved[2] (%)	77.5	72.6	74.8
Reported treating currently stored water (%)	13.4	9.9	13.2
**Sanitation**			
Latrine			
Own any latrine (%)	85.6	83.3	82.0
Access to improved latrine[2] (%)	15.5	16.5	17.4
Always or usually use primary toilet for defecation (%)	96.5	95.0	93.8
Daily defecating in the open, children 0 to <3 years (%)	79.8	76.1	77.4
Human feces observed in compound (%)	7.3	9.2	8.4
**Handwashing**			
Has water within 2 meters of handwashing location (%)	24.1	27.9	26.4
Has soap within 2 meters of handwashing location (%)	9.9	11.1	9.3
**Food security**			
Prevalence of moderate to severe household hunger[3] (%)	11.7	11.3	10.9

[1]Mean ± SD except where noted.

[2]Drinking water and sanitation facilities were considered 'improved' if they met the WHO/UNICEF Joint Monitoring Programme criteria. Improved drinking water was defined as piped water, public tap, tubewell or borehole, protected well, or protected spring. Improved sanitation was defined as flush/pour flush pit latrine, ventilated improved pit latrine, pit latrine with slab, or composting toilet.

[3]Moderate severe hunger defined using the Household Hunger Scale (3).

Supplemental Table 3: Intervention adherence by group in the WASH Benefits Kenya Trial

	Control	WSH	Nutrition	WSH+N
**Number of substudy households assessed**				
Enrolment	644	577	537	542
Year 1	365	320	295	305
Year 2	504	426	420	448
**Visited by promoter in past month**				
Enrolment	-	-	-	-
Year 1	226 (68%)	226 (75%)	234 (84%)	245 (84%)
Year 2	192 (39%)	144 (35%)	171 (42%)	165 (38%)
**Stored drinking water has detectable chlorine**				
Enrolment	13 (3%)	20 (5%)	7 (2%)	14 (3%)
Year 1	9 (3%)	105 (39%)	4 (1%)	102 (41%)
Year 2	6 (1%)	65 (17%)	10 (2%)	79 (19%)
**Access to improved latrine**				
Enrolment	104 (17%)	95 (17%)	73 (14%)	79 (15%)
Year 1	62 (18%)	283 (90%)	41 (15%)	265 (89%)
Year 2	97 (20%)	333 (82%)	66 (16%)	350 (82%)
**Child faeces safely disposed of**				
Enrolment	39 (15%)	41 (17%)	25 (12%)	33 (16%)
Year 1	123 (39%)	182 (66%)	97 (38%)	178 (66%)
Year 2	39 (8%)	107 (28%)	33 (9%)	118 (30%)
**Handwashing location has water and soap**				
Enrolment	32 (5%)	37 (6%)	36 (7%)	34 (6%)
Year 1	37 (10%)	262 (82%)	35 (12%)	241 (79%)
Year 2	40 (8%)	85 (20%)	38 (9%)	103 (23%)
**LNS sachets consumed (% of expected)**				
Enrolment	-	-	-	-
Year 1	-	-	95%	94%
Year 2	-	-	124%	124%

Data are n (%) or %. Free chlorine in drinking water and LNS consumption were not measured at enrolment and were only measured in a subset of groups. LNS adherence measured as proportion of 14 sachets reported consumed in the past week among index children 6-24 months of age.

Supplemental Table 4: Enrollment characteristics by presence in study sample in the WASH Benefits Bangladesh Trial[1]

	Substudy		
	**Included in the analysis**	**Missing data**	**Non-substudy compounds**
No. of compounds:	(N=1464)	(N=575)	(N=3512)
**Maternal**			
Age (years)	24.0 ± 5.2	23.6 ± 5.1	23.7 ± 5.3
Maternal height (cm)	150.4 ± 5.3	150.1 ± 5.5	150.6 ± 5.2
Number of previous births	1.4 ± 1.4	1.6 ± 1.5	1.4 ± 1.4
Completed at least primary (%)	74.0	68.7	69.8
**Paternal**			
Completed at least primary (%)	60.3	57.2	55.9
Works in agriculture (%)	28.8	29.6	32.9
**Household**			
Number of people per compound	11.0 ± 6.2	10.6 ± 6.4	11.1 ± 6.3
Number of people per household	4.8 ± 2.2	4.7 ± 2.3	4.6 ± 2.1
Number of children <18 years in the household	1.6 ± 1.3	1.5 ± 1.3	1.6 ± 1.3
Has electricity (%)	62.6	53.0	58.2
Has a cement floor (%)	12.8	12.5	9.2
Has an iron roof (%)	98.7	98.8	98.4
**Drinking Water**			
Shallow tube well primary water source (%)	73.3	68.5	75.2
Stored water observed at home (%)	47.6	54.1	46.5
Reported treating currently stored water (%)	0.1	0.0	0.2
**Sanitation**			
Own any latrine (%)	55.3	55.3	52.6
Open defication by adult (%)	7.9	7.8	7.9
Open defication by child < 8 years (%)	9.5	8.5	8.3
Human feces observed in house or child play area (%)	8.0	6.4	8.6
**Handwashing**			
Within 6 steps of latrine			
Has water (%)	12.7	11.1	10.1
Has soap (%)	6.7	7.5	5.4
Within 6 steps of kitchen			
Has water (%)	10.0	8.3	7.1
Has soap (%)	3.9	1.2	1.9
**Food security**			
Prevalence of food insecurity[2] (%)	29.3	32.1	31.7

[1]Data are mean ± SD except where noted.

[2]Any level of food insecurity assessed using the Household Food Insecurity Access Scale (4).

Supplemental Table 5: Intervention adherence by group in the WASH Benefits Bangladesh Trial

	Control	WSH	Nutrition	WSH+N
**Number of substudy compounds assessed**				
Enrolment	332	417	410	403
Year 1	278	356	343	350
Year 2	268	347	335	343
**Stored drinking water**				
Enrolment	192 (58%)	196 (47%)	206 (50%)	221 (55%)
Year 1	141 (51%)	345 (97%)	160 (47%)	337 (96%)
Year 2	133 (50%)	323 (93%)	156 (47%)	334 (97%)
**Stored drinking water has detectable chlorine**				
Enrolment	-	-	-	-
Year 1	-	280 (80%)	-	276 (80%)
Year 2	-	274 (81%)	-	290 (86%)
**Latrine with a functional water seal**				
Enrolment	97 (33%)	72 (22%)	92 (28%)	90 (28%)
Year 1	82 (33%)	334 (94%)	76 (25%)	337 (96%)
Year 2	86 (35%)	332 (96%)	93 (31%)	329 (96%)
**No visible faeces on latrine slab or floor**				
Enrolment	151 (48%)	157 (41%)	167 (45%)	162 (44%)
Year 1	174 (66%)	309 (87%)	175 (54%)	311 (89%)
Year 2	148 (58%)	282 (82%)	175 (56%)	286 (84%)
**Handwashing location has soap**				
Enrolment	66 (24%)	84 (22%)	81 (22%)	71 (19%)
Year 1	71 (32%)	321 (90%)	79 (27%)	321 (92%)
Year 2	74 (28%)	308 (89%)	88 (26%)	312 (91%)
**LNS sachets consumed (% of expected)**				
Enrolment	-	-	-	-
Year 1	-	-	92%	92%
Year 2	-	-	92%	90%

Data are n (%) or %. Free chlorine in drinking water and LNS consumption were not measured at enrolment and were only measured in a subset of groups. LNS adherence measured as proportion of 14 sachets reported consumed in the past week among index children 6-24 months of age.

Supplemental Table 6: Prevalence differences between groups in the WASH Benefits Kenya Trial

Outcome	N	Prevalence	Difference vs. Control (95% CI)	Difference vs. WSH (95% CI)	Difference vs. Nutrition (95% CI)
**Anemia**					
(Hb<110 g/L)					
Control	162	48.8	Ref		
WSH	138	45.7	-3.1 (-14.1, 7.9)	Ref	
Nutrition	196	36.2	-12.5 (-22.6, -2.5)*		Ref
WSH+N	154	27.3	-21.5 (-31.4, -11.6)***	-18.4 (-29.7, -7.1)**	-9.0 (-19.7, 1.8)
**Low Hepcidin**					
(Hepcidin<5.5 ng/mL)					
Control	158	48.1	Ref		
WSH	131	50.4	2.3 (-8.2, 12.7)	Ref	
Nutrition	182	17.0	-31.1 (-41.4, -20.7)***		Ref
WSH+N	149	23.5	-24.6 (-35.4, -13.8)***	-26.9 (-37.7, -16.1)***	6.5 (-4.5, 17.4)
**Low Ferritin**					
(FER<12 μg/L)					
Control	157	29.9	Ref		
WSH	125	37.6	7.7 (-2.9, 18.2)	Ref	
Nutrition	173	13.9	-16.1 (-24.6, -7.5)***		Ref
WSH+N	144	9.0	-20.9 (-28.9, -12.9)***	-28.6 (-38.2, -19.0)***	-4.8 (-11.9, 2.2)
**High sTfR**					
(sTfR>8.3 mg/L)					
Control	157	79.6	Ref		
WSH	125	75.2	-4.4 (-15.1, 6.2)	Ref	
Nutrition	173	54.3	-25.3 (-33.7, -16.8)***		Ref
WSH+N	144	61.1	-18.5 (-29.6, -7.5)**	-14.1 (-25.4, -2.8)*	6.8 (-4.0, 17.6)
**Iron Deficiency**					
(FER<12 μg/L or sTfR>8.3 mg/L)					
Control	157	80.9	Ref		
WSH	125	77.6	-3.3 (-14.1, 7.5)	Ref	
Nutrition	173	57.2	-23.7 (-32.1, -15.3)***		Ref
WSH+N	144	62.5	-18.4 (-29.0, -7.8)***	-15.1 (-25.8, -4.4)**	5.3 (-4.9, 15.4)
**Iron Deficiency Anemia**					
(anemic and iron deficient)					
Control	152	40.8	Ref		
WSH	131	38.2	-2.6 (-12.4, 7.2)	Ref	
Nutrition	189	23.8	-17.0 (-26.6, -7.3)***		Ref
WSH+N	154	20.1	-20.7 (-30.9, -10.5)***	-18.0 (-28.8, -7.2)**	-3.7 (-13.4, 6.0)
**Vitamin A Deficiency**					
(RBP<0.83 μmol/L)					
Control	157	52.9	Ref		
WSH	125	44.0	-8.9 (-22.3, 4.6)	Ref	
Nutrition	173	34.7	-18.2 (-30.2, -6.1)**		Ref
WSH+N	144	28.5	-24.4 (-36.0, -12.8)***	-15.5 (-28.7, -2.4)*	-6.2 (-16.8, 4.3)
**B12 Deficiency**					
(B12<150 pmol/L)					
Control	156	4.5	Ref		
WSH	126	5.6	1.1 (-5.1, 7.2)	Ref	
Nutrition	178	3.9	-0.6 (-3.9, 2.8)		Ref
WSH+N	142	2.8	-1.7 (-6.0, 2.6)	-2.7 (-8.5, 3.0)	-1.1 (-4.7, 2.4)
**B12 Depletion or Deficiency**					
(B12<221 pmol/L)					
Control	156	21.8	Ref		
WSH	126	21.4	-0.4 (-9.8, 9.1)	Ref	
Nutrition	178	12.9	-8.9 (-16.1, -1.6)*		Ref
WSH+N	142	9.9	-11.9 (-20.1, -3.8)**	-11.6 (-20.6, -2.6)*	-3.1 (-9.3, 3.2)
**Folate Deficiency**					
(Folate<10 nmol/L)					
Control	156	9.6	Ref		
WSH	129	14.0	4.3 (-4.4, 13.1)	Ref	
Nutrition	178	1.1	-8.5 (-14.1, -2.9)**		Ref
WSH+N	142	0.7	-8.9 (-14.0, -3.8)***	-13.2 (-20.0, -6.5)***	-0.4 (-2.5, 1.7)
**High Folate**					
(Folate>45.3 nmol/L)					
Control	156	3.2	Ref		
WSH	129	7.8	4.5 (-1.1, 10.2)	Ref	
Nutrition	178	42.1	38.9 (30.1, 47.8)***		Ref
WSH+N	142	35.9	32.7 (23.8, 41.6)***	28.2 (19.3, 37.0)***	-6.2 (-18.2, 5.8)

* p<0.05, ** p<0.01, *** p<0.001

Supplemental Table 7: Prevalence ratio for iron deficiency, iron deficiency anemia, and vitamin A deficiency after correcting values for inflammation and malaria status, comparing each intervention group to the control in the WASH Benefits Kenya Trial

**Outcome**	**N**	**Prevalence**	**Ratio vs. Control (95% CI)**
**High AGP**			
(AGP>1 g/L)			
Control	157	49.7	Ref
WSH	125	44.0	0.89 (0.67, 1.18)
Nutrition	173	43.9	0.88 (0.72, 1.08)
WSH+N	144	49.3	0.99 (0.79, 1.25)
**High CRP**			
(CRP>5 mg/L)			
Control	157	22.9	Ref
WSH	125	17.6	0.77 (0.45, 1.30)
Nutrition	173	22.0	0.96 (0.59, 1.55)
WSH+N	144	20.1	0.88 (0.54, 1.43)
**Malaria**			
Control	164	18.9	Ref
WSH	139	15.8	0.84 (0.53, 1.33)
Nutrition	196	19.4	1.03 (0.65, 1.62)
WSH+N	155	21.9	1.16 (0.66, 2.05)
**Low Ferritin**			
(FER<12 μg/L)			
Control	144	58.3	Ref
WSH	115	58.3	1.00 (0.79, 1.27)
Nutrition	166	24.7	0.42 (0.30, 0.60)***
WSH+N	132	22.0	0.38 (0.26, 0.55)***
**High sTfR**			
(sTfR>8.3 mg/L)			
Control	144	72.9	Ref
WSH	115	73.0	1.00 (0.86, 1.17)
Nutrition	166	47.6	0.65 (0.55, 0.77)***
WSH+N	132	53.8	0.74 (0.61, 0.89)**
**Iron Deficiency**			
(FER<12 μg/L or sTfR>8.3 mg/L)			
Control	144	84.7	Ref
WSH	115	79.1	0.93 (0.83, 1.06)
Nutrition	166	53.6	0.63 (0.55, 0.73)***
WSH+N	132	59.1	0.70 (0.60, 0.81)***
**Iron Deficiency Anemia**			
(anemic and iron deficient)			
Control	152	42.1	Ref
WSH	129	38.8	0.92 (0.73, 1.17)
Nutrition	186	20.4	0.49 (0.36, 0.65)***
WSH+N	148	17.6	0.42 (0.28, 0.63)***
**Vitamin A Deficiency**			
(RBP<0.83 μmol/L)			
Control	144	33.3	Ref
WSH	115	23.5	0.70 (0.49, 1.01)
Nutrition	166	9.0	0.27 (0.15, 0.48)***
WSH+N	132	11.4	0.34 (0.20, 0.58)***

* p<0.05, ** p<0.01, *** p<0.001

Supplemental Table 8: Comparison of mean differences between intervention groups after adjusting for covariates[1] and using inverse probability of censoring weighted (IPCW) analysis in the WASH Benefits Kenya Trial

Outcome	N	Median (Q1, Q3)	Adjusted Comparison vs. Control (95% CI)	IPCW Comparison vs. Control (95% CI)
			**Difference**	**Difference**
**Hemoglobin (g/L)**				
Control	159	111 (104, 118)	Ref	Ref
WSH	132	111 (102, 118)	-1.1 (-3.2, 1.1)	-0.7 (-3.2, 1.9)
Nutrition	192	115 (106, 122)	2.8 (-0.3, 6.0)	2.9 (-0.2, 5.9)
WSH+N	151	116 (108, 122)	5.1 (2.2, 8.0)***	4.5 (1.7, 7.4)**
			**Percent Difference**	**Percent Difference**
**RBP (μmol/L)[2]**				
Control	153	0.81 (0.65, 1.02)	Ref	Ref
WSH	119	0.85 (0.69, 1.04)	6.2 (-1.6, 14.6)	4.1 (-4.4, 13.3)
Nutrition	169	0.91 (0.77, 1.09)	16.5 (7.7, 25.9)***	15.0 (6.1, 24.6)***
WSH+N	141	0.93 (0.78, 1.08)	16.4 (8.1, 25.4)***	15.3 (6.6, 24.6)***
**Ferritin (μg/L)[2]**				
Control	153	18.0 (10.3, 30.8)	Ref	Ref
WSH	119	15.5 (8.9, 30.1)	-15.1 (-28.5, 0.8)	-10.1 (-26.6, 10.1)
Nutrition	169	31.8 (19.8, 51.5)	71.7 (46.8, 101.0)***	71.9 (38.9, 112.6)***
WSH+N	141	32.4 (20.5, 49.1)	49.5 (24.4, 79.6)***	59.8 (32.3, 93.0)***
**sTfR (mg/L)[2]**				
Control	153	12.1 (8.8, 17.1)	Ref	Ref
WSH	119	12.2 (8.4, 18.6)	3.0 (-5.2, 12.0)	0.4 (-8.8, 10.5)
Nutrition	169	8.5 (7.3, 12.4)	-22.5 (-29.1, -15.2)***	-23.2 (-29.8, -15.9)***
WSH+N	141	9.6 (7.3, 12.8)	-22.2 (-27.9, -16.0)***	-21.3 (-28.5, -13.3)***
**Hepcidin (ng/mL)[2]**				
Control	154	6.1 (2.3, 13.5)	Ref	Ref
WSH	125	4.6 (1.5, 10.3)	-28.0 (-46.7, -2.7)*	-30.2 (-48.9, -4.6)*
Nutrition	178	13.0 (6.8, 22.2)	110.1 (50.1, 194.1)***	113.9 (56.1, 193.2)***
WSH+N	146	14.3 (6.8, 22.6)	106.0 (50.1, 182.9)***	93.9 (34.1, 180.5)***
**B12 (pmol/L)[2]**				
Control	152	299.5 (225.5, 413.2)	Ref	Ref
WSH	120	305.4 (233.7, 411.6)	-1.6 (-11.8, 9.8)	-3.4 (-14.0, 8.4)
Nutrition	174	362.2 (273.2, 531.3)	20.3 (9.2, 32.6)***	19.8 (8.6, 32.1)***
WSH+N	140	371.6 (282.8, 515.4)	19.9 (6.2, 35.5)**	21.5 (8.3, 36.3)***

* p<0.05, ** p<0.01, *** p<0.001

[1]Covariates considered in all analyses included child age, sex, birth order, maternal age, height, education level, household hunger score, number of children <18 y in the household, number of individuals living in the compound, distance to primary water source, housing materials, household assets, animal ownership, month of measurement, malaria infection, and either sickle cell or thalassemia trait.

[2]Log transformed for analysis.

Supplemental Table 9: Effect modification with genetic trait in the WASH Benefits Kenya Trial

	No genetic trait			Has genetic trait[2]			
**Outcome**	**N**	**Median (Q1, Q3)**	**Comparison (95% CI)**	**N**	**Median (Q1, Q3)**	**Comparison (95% CI)**	**P for Interaction**
			**Difference**			**Difference**	
**Hemoglobin (g/L)**							
Control	45	114 (108, 122)		61	106 (97, 116)		
WSH	45	111 (100, 122)	-4.6 (-9.9, 0.6)	45	111 (99, 116)	3.2 (-1.1, 7.5)	0.017
Nutrition	56	119 (108, 125)	2.2 (-3.2, 7.7)	83	113 (105, 120)	7.3 (3.2, 11.5)	0.111
WSH+N	51	116 (109, 124)	1.3 (-3.9, 6.4)	65	115 (107, 120)	8.6 (3.5, 13.7)	0.014
WSH+N vs WSH			5.9 (1.4, 10.4)			5.4 (0.9, 10.0)	0.886
WSH+N vs N			-1.0 (-6.0, 4.0)			1.3 (-3.1, 5.6)	0.491
			**Percent Difference**			**Percent Difference**	
**RBP (μmol/L)**							
Control	45	0.8 (0.7, 1.0)		56	0.8 (0.6, 1.0)		
WSH	40	0.8 (0.7, 1.0)	1.1 (-11.3, 15.2)	41	0.9 (0.7, 1.0)	10.8 (-3.9, 27.7)	0.352
Nutrition	51	0.9 (0.7, 1.1)	10.1 (-6.2, 29.1)	70	0.9 (0.8, 1.1)	19.3 (7.4, 32.6)	0.403
WSH+N	53	0.9 (0.7, 1.1)	9.8 (-2.0, 23.0)	61	0.9 (0.8, 1.0)	18.5 (4.6, 34.1)	0.374
WSH+N vs WSH			8.6 (-2.9, 21.5)			6.9 (-6.0, 21.5)	0.824
WSH+N vs N			-0.2 (-11.5, 12.4)			-0.7 (-8.8, 8.1)	0.938
**Ferritin (μg/L)[1]**							
Control	45	15.3 (8.9, 27.0)		56	21.0 (8.7, 43.1)		
WSH	40	12.3 (8.6, 27.4)	-11.8 (-40.0, 29.6)	41	18.4 (9.9, 38.0)	-11.5 (-43.1, 37.8)	0.989
Nutrition	51	33.9 (23.7, 77.8)	129.5 (68.2, 213.1)	70	31.4 (22.4, 47.9)	40.0 (-1.7, 99.3)	0.053
WSH+N	53	37.6 (25.6, 58.0)	111.7 (49.5, 199.9)	61	27.7 (19.0, 38.6)	29.5 (-10.6, 87.8)	0.070
WSH+N vs WSH			140.2 (54.7, 272.7)			46.3 (8.8, 96.7)	0.049
WSH+N vs N			-7.7 (-32.8, 26.7)			-7.5 (-26.1, 15.8)	0.989
**sTfR (mg/L)[1]**							
Control	45	11.6 (9.8, 15.4)		56	15.4 (11.2, 19.4)		
WSH	40	12.6 (9.0, 19.7)	12.8 (-2.9, 31.0)	41	14.1 (8.8, 18.7)	-14.5 (-27.9, 1.3)	0.029
Nutrition	51	8.4 (7.3, 11.8)	-22.6 (-33.0, -10.5)	70	8.6 (7.4, 12.9)	-37.0 (-46.1, -26.4)	0.058
WSH+N	53	9.9 (7.2, 13.7)	-11.6 (-25.7, 5.0)	61	10.1 (7.9, 12.8)	-33.8 (-43.0, -23.1)	0.013
WSH+N vs WSH			-21.7 (-34.0, -7.0)			-22.5 (-33.4, -9.9)	0.921
WSH+N vs N			14.1 (-1.5, 32.1)			5.1 (-8.7, 21.0)	0.451
**Hepcidin (ng/mL)[1]**							
Control	44	5.6 (2.5, 11.3)		56	3.4 (1.5, 11.4)		
WSH	41	3.3 (1.1, 7.2)	-46.0 (-72.6, 6.3)	43	4.4 (1.5, 10.0)	-13.0 (-55.2, 68.7)	0.296
Nutrition	51	14.6 (6.6, 23.5)	153.0 (36.6, 368.7)	76	13.6 (7.8, 22.7)	195.5 (98.5, 340.0)	0.639
WSH+N	52	15.4 (6.9, 24.6)	126.6 (14.4, 348.9)	63	12.2 (7.9, 16.9)	134.8 (60.9, 242.5)	0.921
WSH+N vs WSH			320.0 (100.5, 780.0)			169.9 (36.6, 433.1)	0.349
WSH+N vs N			-10.4 (-45.2, 46.4)			-20.5 (-42.6, 10.0)	0.671
**B12 (pmol/L)[1]**							
Control	44	314.8 (239.4, 416.5)		57	263.2 (215.9, 405.6)		
WSH	40	353.0 (265.1, 444.3)	0.1 (-14.2, 16.8)	42	262.1 (196.8, 353.5)	-10.9 (-24.6, 5.3)	0.245
Nutrition	53	376.7 (279.0, 559.3)	23.1 (2.2, 48.2)	73	339.1 (254.9, 459.7)	12.8 (-4.2, 32.8)	0.483
WSH+N	51	364.3 (279.6, 445.4)	13.7 (-4.7, 35.6)	60	397.5 (293.8, 559.0)	35.6 (14.9, 60.1)	0.116
WSH+N vs WSH			13.6 (-4.8, 35.4)			52.2 (31.4, 76.2)	0.020
WSH+N vs N			-7.6 (-24.4, 12.8)			20.2 (5.2, 37.4)	0.015

[1]Log transformed for analysis.

[2]Presence of sickle cell or thalassemia trait.

Supplemental Table 10: Prevalence differences between groups in the WASH Benefits Bangladesh Trial

Outcome	N	Prevalence	Difference vs. Control (95% CI)	Difference vs. WSH (95% CI)	Difference vs. Nutrition (95% CI)
**Anemia**					
(Hb<110 g/L)					
Control	340	17.4	Ref		
WSH	384	12.8	-4.6 (-9.4, 0.2)	Ref	
Nutrition	356	8.7	-8.6 (-14.2, -3.1)**		Ref
WSH+N	390	7.9	-9.4 (-15.1, -3.7)**	-4.8 (-8.7, -0.9)*	-0.8 (-4.7, 3.2)
**Low Hepcidin**					
(Hepcidin<5.5 ng/mL)					
Control	156	23.7	Ref		
WSH	217	20.7	-3.0 (-11.8, 5.8)	Ref	
Nutrition	178	9.6	-14.2 (-22.8, -5.5)**		Ref
WSH+N	181	6.6	-17.1 (-24.7, -9.5)***	-14.1 (-20.4, -7.8)***	-2.9 (-8.4, 2.5)
**Low Ferritin**					
(FER<12 μg/L)					
Control	310	20.0	Ref		
WSH	370	15.4	-4.6 (-10.2, 1.0)	Ref	
Nutrition	336	5.4	-14.6 (-19.7, -9.6)***		Ref
WSH+N	372	3.0	-17.0 (-21.5, -12.6)***	-12.4 (-16.2, -8.7)***	-2.4 (-5.3, 0.5)
**High sTfR**					
(sTfR>8.3 mg/L)					
Control	310	27.1	Ref		
WSH	370	26.8	-0.3 (-7.8, 7.1)	Ref	
Nutrition	336	12.5	-14.6 (-22.2, -7.0)***		Ref
WSH+N	372	13.2	-13.9 (-21.3, -6.6)***	-13.6 (-19.0, -8.1)***	0.7 (-5.3, 6.6)
**Iron Deficiency**					
(FER<12 μg/L or sTfR>8.3 mg/L)					
Control	310	34.8	Ref		
WSH	370	32.2	-2.7 (-10.5, 5.2)	Ref	
Nutrition	336	15.8	-19.1 (-27.0, -11.2)***		Ref
WSH+N	372	14.8	-20.1 (-27.9, -12.2)***	-17.4 (-23.4, -11.3)***	-1.0 (-6.9, 4.9)
**Iron Deficiency Anemia**					
(anemic and iron deficient)					
Control	329	9.7	Ref		
WSH	378	6.6	-3.1 (-7.9, 1.6)	Ref	
Nutrition	351	2.6	-7.2 (-12.0, -2.3)**		Ref
WSH+N	387	1.3	-8.4 (-12.9, -4.0)***	-5.3 (-8.0, -2.7)***	-1.3 (-3.4, 0.9)
**Vitamin A Deficiency**					
(RBP<0.83 μmol/L)					
Control	310	16.1	Ref		
WSH	370	19.7	3.6 (-3.2, 10.4)	Ref	
Nutrition	336	16.7	0.5 (-6.9, 7.9)		Ref
WSH+N	372	11.8	-4.3 (-10.1, 1.5)	-7.9 (-13.5, -2.3)**	-4.8 (-10.3, 0.6)
**B12 Deficiency**					
(B12<150 pmol/L)					
Control	304	2.3	Ref		
WSH	360	3.1	0.8 (-1.9, 3.4)	Ref	
Nutrition	332	3.0	0.7 (-2.4, 3.9)		Ref
WSH+N	363	1.4	-0.9 (-3.3, 1.4)	-1.7 (-4.1, 0.7)	-1.6 (-4.4, 1.1)
**B12 Depletion or Deficiency**					
(B12<221 pmol/L)					
Control	304	20.7	Ref		
WSH	360	16.1	-4.6 (-10.9, 1.7)	Ref	
Nutrition	332	11.4	-9.3 (-16.0, -2.5)**		Ref
WSH+N	363	16.3	-4.5 (-10.3, 1.4)	0.1 (-5.3, 5.6)	4.8 (-1.0, 10.6)
**Folate Deficiency**					
(Folate<10 nmol/L)					
Control	304	2.3	Ref		
WSH	359	5.8	3.5 (0.4, 6.7)*	Ref	
Nutrition	329	2.7	0.4 (-2.0, 2.8)		Ref
WSH+N	363	2.8	0.5 (-1.7, 2.6)	-3.1 (-6.1, -0.1)*	0.0 (-2.5, 2.6)
**High Folate**					
(Folate>45.3 nmol/L)					
Control	304	9.2	Ref		
WSH	359	8.1	-1.1 (-6.5, 4.2)	Ref	
Nutrition	329	8.5	-0.7 (-6.5, 5.1)		Ref
WSH+N	363	7.4	-1.8 (-6.8, 3.2)	-0.6 (-4.2, 2.9)	-1.1 (-5.2, 3.0)

* p<0.05, ** p<0.01, *** p<0.001

Supplemental Figure 2: Mean serum folate concentration by study group over the intervention period in the WASH Benefits Bangladesh Trial

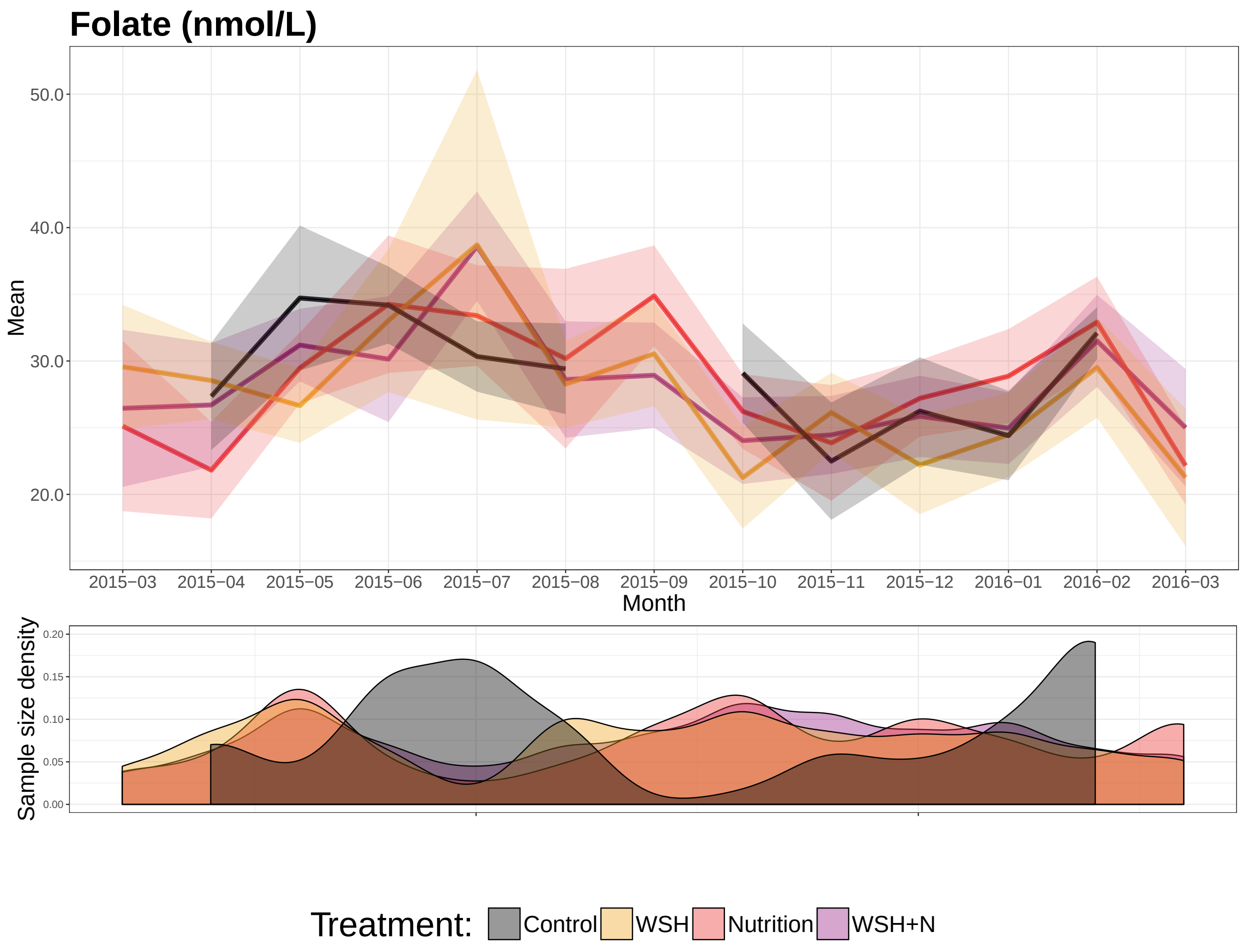

Supplemental Table 11: Comparison of mean differences between intervention groups: unadjusted, after adjusting for covariates[1], and using inverse probability of censoring weighted (IPCW) analysis in the WASH Benefits Bangladesh Trial

Outcome	N	Median (Q1, Q3)	Unadjusted Comparison vs. Control (95% CI)	Adjusted Comparison vs. Control (95% CI)	IPCW Comparison vs. Control (95% CI)
			**Difference**	**Difference**	**Difference**
**Hemoglobin (g/L)**					
Control	333	119 (113, 125)	Ref	Ref	Ref
WSH	376	119 (114, 125)	0.5 (-1.0, 2.0)	1.0 (-0.4, 2.5)	0.5 (-1.2, 2.2)
Nutrition	351	121 (116, 127)	2.7 (1.1, 4.3)***	2.0 (-0.3, 4.3)	2.6 (0.5, 4.8)*
WSH+N	387	121 (116, 127)	2.4 (0.9, 4.0)**	2.0 (0.5, 3.6)**	2.2 (0.8, 3.6)**
			**Percent Difference**	**Percent Difference**	**Percent Difference**
**RBP (μmol/L)[2]**					
Control	305	1.07 (0.91, 1.28)	Ref	Ref	Ref
WSH	362	1.05 (0.86, 1.25)	-3.1 (-8.4, 2.5)	2.8 (-2.5, 8.4)	1.5 (-2.9, 6.1)
Nutrition	331	1.09 (0.90, 1.28)	0.1 (-5.3, 5.9)	4.0 (-1.1, 9.4)	1.7 (-3.3, 7.0)
WSH+N	369	1.12 (0.94, 1.30)	2.2 (-2.7, 7.4)	8.1 (3.7, 12.7)***	5.7 (1.6, 10.0)**
**Folate (nmol/L)[2]**					
Control	299	30.2 (22.5, 37.5)	Ref	Ref	Ref
WSH	351	26.0 (17.9, 34.0)	-13.9 (-20.6, -6.5)***	-3.9 (-10.5, 3.2)	-8.5 (-15.2, -1.2)*
Nutrition	324	27.8 (19.7, 35.5)	-7.5 (-15.5, 1.2)	0.9 (-7.1, 9.7)	-1.2 (-8.9, 7.1)
WSH+N	360	26.8 (19.6, 35.4)	-8.7 (-15.4, -1.4)*	-2.0 (-9.0, 5.7)	-5.1 (-11.3, 1.5)
**Ferritin (μg/L)[2]**					
Control	305	24.2 (14.1, 37.4)	Ref	Ref	Ref
WSH	362	25.4 (14.6, 40.9)	5.9 (-5.5, 18.7)	10.9 (-2.3, 25.8)	9.3 (-2.8, 22.9)
Nutrition	331	37.9 (25.7, 52.0)	56.2 (40.9, 73.2)***	48.5 (32.7, 66.0)***	57.5 (30.3, 90.5)***
WSH+N	369	39.3 (26.2, 53.8)	63.6 (45.8, 83.5)***	60.8 (46.0, 77.0)***	63.8 (50.3, 78.5)***
**sTfR (mg/L)[2]**					
Control	305	7.2 (6.1, 8.5)	Ref	Ref	Ref
WSH	362	7.1 (6.0, 8.4)	0.0 (-0.1, 0.0)	0.0 (-0.1, 0.0)	0.0 (-0.1, 0.1)
Nutrition	331	6.5 (5.9, 7.5)	-0.1 (-0.2, -0.1)***	-0.1 (-0.2, -0.1)***	-0.1 (-0.2, 0.0)***
WSH+N	369	6.7 (6.0, 7.6)	-0.1 (-0.2, -0.1)***	-0.1 (-0.2, -0.1)***	-0.1 (-0.2, -0.1)***
**Hepcidin (ng/mL)[2]**					
Control	154	13.8 (6.1, 24.4)	Ref	Ref	Ref
WSH	209	13.1 (6.4, 26.0)	-5.7 (-29.3, 25.9)	5.5 (-16.2, 32.8)	1.9 (-18.2, 27.0)
Nutrition	174	16.7 (9.5, 27.9)	48.8 (15.7, 91.6)**	33.1 (1.2, 75.0)*	62.7 (29.8, 103.8)***
WSH+N	179	19.1 (11.8, 31.5)	64.4 (30.6, 107.1)***	53.4 (20.3, 95.8)***	69.2 (43.7, 99.2)***
**B12 (pmol/L)[2]**					
Control	299	296.4 (238.1, 395.5)	Ref	Ref	Ref
WSH	352	312.2 (247.2, 418.6)	5.1 (-2.1, 12.7)	2.8 (-3.8, 9.8)	4.3 (-2.1, 11.1)
Nutrition	327	348.1 (275.8, 436.8)	11.6 (3.8, 20.0)**	7.1 (-1.9, 16.9)	10.5 (0.3, 21.8)*
WSH+N	360	344.7 (250.5, 447.1)	11.8 (4.5, 19.7)**	12.1 (4.6, 20.1)**	12.6 (4.9, 21.0)**

* $p<0.05$, ** $p<0.01$, *** $p<0.001$

[1]Covariates considered in all analyses included child age, sex, birth order, maternal age, height, education level, household food security score, number of children <18 y in the household, number of individuals living in the compound, distance to primary water source, tubewell water source, housing materials, household assets, animal ownership, month of measurement, and either sickle cell or thalassemia trait.

[2]Log transformed for analysis.

Supplemental Table 12: Prevalence ratio for iron deficiency, iron deficiency anemia, and Vitamin A deficiency after correcting values for inflammation, comparing each intervention group to the control in the WASH Benefits Bangladesh Trial

Outcome	N	Prevalence	Ratio vs. Control (95% CI)
**High CRP**			
(CRP>5 mg/L)			
Control	310	8.7	Ref
WSH	370	10.8	1.24 (0.77, 1.99)
Nutrition	336	10.7	1.23 (0.72, 2.10)
WSH+N	372	11.3	1.30 (0.80, 2.09)
**Low Ferritin**			
(FER<12 µg/L)			
Control	310	29.7	Ref
WSH	370	27.3	0.92 (0.73, 1.16)
Nutrition	336	8.3	0.28 (0.18, 0.43)***
WSH+N	372	6.5	0.22 (0.15, 0.32)***
**High sTfR**			
(sTfR>8.3 mg/L)			
Control	310	26.8	Ref
WSH	370	26.2	0.98 (0.75, 1.28)
Nutrition	336	11.9	0.44 (0.30, 0.66)***
WSH+N	372	11.8	0.44 (0.30, 0.65)***
**Iron Deficiency**			
(FER<12 µg/L or sTfR>8.3 mg/L)			
Control	310	41.0	Ref
WSH	370	38.4	0.94 (0.78, 1.12)
Nutrition	336	17.0	0.41 (0.31, 0.55)***
WSH+N	372	15.6	0.38 (0.28, 0.52)***
**Iron Deficiency Anemia**			
(anemic and iron deficient)			
Control	329	10.0	Ref
WSH	378	7.7	0.76 (0.45, 1.29)
Nutrition	351	2.6	0.26 (0.11, 0.60)**
WSH+N	387	1.3	0.13 (0.05, 0.34)***
**Vitamin A Deficiency**			
(RBP<0.83 µmol/L)			
Control	310	6.1	Ref
WSH	370	12.2	1.98 (1.13, 3.50)*
Nutrition	336	7.4	1.21 (0.60, 2.45)
WSH+N	372	4.0	0.66 (0.31, 1.38)

* $p<0.05$, ** $p<0.01$, *** $p<0.001$

Supplemental Table 13: Effect modification with child age in the WASH Benefits Bangladesh Trial

	28m or younger			Over 28m			
**Outcome**	**N**	**Median (Q1, Q3)**	**Comparison (95% CI)**	**N**	**Median (Q1, Q3)**	**Comparison (95% CI)**	**P for Interaction**
			**Difference**			**Difference**	
**Hemoglobin (g/L)**							
Control	117	119 (113, 125)		220	119 (112, 125)		
WSH	202	119 (114, 125)	0.2 (-2.5, 2.9)	182	120 (114, 127)	1.1 (-0.8, 2.9)	0.606
Nutrition	163	122 (116, 127)	2.9 (0.4, 5.4)	193	121 (116, 127)	2.7 (0.9, 4.4)	0.856
WSH+N	207	121 (116, 126)	2.3 (-0.2, 4.8)	183	122 (116, 127)	2.8 (0.9, 4.7)	0.769
WSH+N vs WSH			2.1 (0.2, 4.1)			1.7 (0.1, 3.3)	0.744
WSH+N vs N			-0.6 (-2.3, 1.2)			0.1 (-1.8, 2.0)	0.595
			**Percent Difference**			**Percent Difference**	
**RBP (μmol/L)[1]**							
Control	113	1.0 (0.8, 1.2)		195	1.1 (0.9, 1.3)		
WSH	193	1.1 (0.9, 1.2)	3.4 (-3.2, 10.5)	177	1.0 (0.8, 1.2)	-7.3 (-13.2, -0.8)	0.008
Nutrition	149	1.0 (0.9, 1.2)	2.8 (-3.8, 10.0)	187	1.1 (0.9, 1.3)	-0.7 (-7.0, 6.0)	0.366
WSH+N	194	1.1 (0.9, 1.3)	6.8 (1.0, 12.9)	178	1.1 (0.9, 1.3)	0.2 (-5.9, 6.8)	0.087
WSH+N vs WSH			3.2 (-2.0, 8.8)			8.1 (3.0, 13.4)	0.190
WSH+N vs N			3.8 (-2.6, 10.6)			1.0 (-3.2, 5.3)	0.426
**Folate (nmol/L)[1]**							
Control	109	30.6 (21.3, 37.4)		193	29.7 (22.9, 37.6)		
WSH	186	27.1 (17.9, 37.2)	-10.8 (-20.2, -0.4)	173	25.4 (18.4, 32.8)	-16.6 (-25.4, -6.8)	0.374
Nutrition	149	30.8 (21.1, 37.7)	0.3 (-12.3, 14.7)	180	26.2 (18.8, 32.0)	-13.4 (-22.0, -3.9)	0.063
WSH+N	190	28.1 (20.3, 37.3)	-5.1 (-15.3, 6.4)	173	25.6 (18.9, 33.8)	-12.0 (-19.8, -3.5)	0.276
WSH+N vs WSH			6.4 (-3.9, 18.0)			5.5 (-3.4, 15.1)	0.886
WSH+N vs N			-5.3 (-14.5, 4.8)			1.6 (-6.3, 10.1)	0.295
**Ferritin (μg/L)[1]**							
Control	113	21.2 (12.5, 34.3)		195	27.1 (15.8, 39.0)		
WSH	193	24.1 (14.3, 40.4)	14.9 (-5.5, 39.7)	177	25.6 (16.9, 42.8)	3.8 (-7.2, 16.2)	0.355
Nutrition	149	37.7 (23.6, 53.1)	75.2 (47.6, 108.1)	187	37.9 (26.2, 51.7)	47.3 (31.0, 65.6)	0.093
WSH+N	194	39.6 (26.1, 54.2)	86.1 (52.0, 127.8)	178	38.4 (26.7, 53.1)	52.3 (35.5, 71.1)	0.084
WSH+N vs WSH			61.9 (40.4, 86.8)			46.7 (30.3, 65.2)	0.287
WSH+N vs N			6.2 (-7.6, 22.0)			3.4 (-8.1, 16.2)	0.761
**sTfR (mg/L)[1]**							
Control	113	7.8 (6.6, 9.2)		195	6.9 (5.9, 8.2)		
WSH	193	7.0 (6.0, 8.3)	-11.7 (-21.2, -1.1)	177	7.2 (6.1, 8.5)	5.0 (-0.7, 11.1)	0.004
Nutrition	149	6.6 (5.9, 7.4)	-21.6 (-29.4, -13.0)	187	6.5 (5.9, 7.6)	-6.6 (-12.1, -0.7)	0.001
WSH+N	194	6.7 (5.9, 7.6)	-20.7 (-28.3, -12.3)	178	6.7 (6.0, 7.6)	-4.7 (-9.7, 0.6)	0.001
WSH+N vs WSH			-10.1 (-14.8, -5.2)			-9.3 (-14.3, -3.9)	0.823
WSH+N vs N			1.2 (-4.1, 6.9)			2.0 (-4.1, 8.4)	0.855
**Hepcidin (ng/mL)[1]**							
Control	53	10.5 (4.4, 17.3)		103	13.9 (7.2, 25.3)		
WSH	106	12.2 (5.3, 22.7)	7.8 (-31.5, 69.5)	111	13.3 (7.3, 27.7)	-10.2 (-41.4, 37.7)	0.578
Nutrition	77	18.3 (9.2, 31.1)	75.0 (10.6, 176.9)	101	16.7 (9.8, 26.5)	37.5 (4.1, 81.5)	0.367
WSH+N	99	21.0 (12.0, 31.9)	105.3 (32.7, 217.8)	82	17.2 (11.9, 30.3)	42.2 (3.0, 96.4)	0.214
WSH+N vs WSH			90.5 (35.3, 168.2)			58.3 (7.6, 132.8)	0.494
WSH+N vs N			17.4 (-12.6, 57.6)			3.4 (-21.8, 36.8)	0.583
**B12 (pmol/L)[1]**							
Control	110	288.7 (241.9, 425.7)		192	300.9 (237.9, 386.4)		
WSH	185	311.1 (250.1, 424.7)	3.2 (-7.2, 14.8)	175	320.7 (252.2, 429.3)	6.8 (-1.6, 15.8)	0.569
Nutrition	149	338.7 (278.9, 428.5)	11.1 (0.0, 23.4)	183	350.7 (274.9, 448.7)	11.8 (2.2, 22.3)	0.921
WSH+N	190	320.2 (250.5, 440.5)	9.4 (-1.2, 21.2)	173	365.8 (254.4, 472.8)	14.2 (3.6, 25.8)	0.542
WSH+N vs WSH			6.1 (-1.1, 13.8)			7.0 (-1.9, 16.6)	0.886
WSH+N vs N			-1.5 (-8.9, 6.6)			2.2 (-7.2, 12.4)	0.546

[1]Log transformed for analysis.

Supplemental Table 14: Effect modification with food security in the WASH Benefits Bangladesh Trial

	Food insecure			Food secure			
**Outcome**	**N**	**Median (Q1, Q3)**	**Comparison (95% CI)**	**N**	**Median (Q1, Q3)**	**Comparison (95% CI)**	**P for Interaction**
			**Difference**			**Difference**	
**Hemoglobin (g/L)**							
Control	89	117 (112, 123)		251	120 (113, 126)		
WSH	127	120 (114, 125)	2.5 (0.2, 4.8)	257	119 (114, 126)	-0.3 (-2.1, 1.5)	0.060
Nutrition	103	120 (116, 126)	3.3 (0.9, 5.6)	253	122 (116, 128)	2.5 (0.7, 4.3)	0.579
WSH+N	112	121 (116, 126)	3.3 (1.2, 5.5)	278	121 (116, 127)	2.1 (0.4, 3.9)	0.365
WSH+N vs WSH			0.8 (-1.1, 2.8)			2.4 (0.9, 3.9)	0.208
WSH+N vs N			0.1 (-1.9, 2.0)			-0.4 (-1.9, 1.2)	0.714
			**Percent Difference**			**Percent Difference**	
**RBP (μmol/L)[1]**							
Control	82	1.1 (0.9, 1.2)		228	1.1 (0.9, 1.3)		
WSH	121	1.0 (0.8, 1.2)	-7.9 (-14.7, -0.5)	249	1.1 (0.9, 1.3)	-0.7 (-6.9, 6.0)	0.098
Nutrition	98	1.1 (0.9, 1.2)	-1.3 (-8.9, 6.9)	238	1.1 (0.9, 1.3)	0.8 (-5.4, 7.3)	0.639
WSH+N	108	1.1 (0.9, 1.3)	0.2 (-7.4, 8.4)	264	1.1 (0.9, 1.3)	3.1 (-2.3, 8.7)	0.508
WSH+N vs WSH			8.8 (0.4, 18.0)			3.8 (-0.8, 8.5)	0.337
WSH+N vs N			1.6 (-5.9, 9.6)			2.3 (-2.7, 7.6)	0.884
**Folate (nmol/L)[1]**							
Control	81	29.8 (24.2, 37.6)		223	30.2 (22.0, 37.0)		
WSH	117	24.4 (16.8, 31.7)	-19.9 (-29.2, -9.5)	242	26.8 (19.6, 35.5)	-10.9 (-18.9, -2.1)	0.122
Nutrition	97	26.0 (19.7, 33.9)	-10.9 (-20.9, 0.3)	232	28.1 (20.0, 36.2)	-6.1 (-15.3, 4.0)	0.435
WSH+N	105	25.4 (18.9, 34.2)	-12.7 (-22.8, -1.3)	258	27.3 (19.9, 35.7)	-7.0 (-15.0, 1.6)	0.389
WSH+N vs WSH			9.0 (-4.0, 23.9)			4.3 (-4.2, 13.6)	0.554
WSH+N vs N			-2.0 (-11.7, 8.8)			-1.0 (-8.5, 7.2)	0.877
**Ferritin (μg/L)[1]**							
Control	82	23.6 (13.6, 38.8)		228	24.4 (14.1, 37.2)		
WSH	121	25.0 (14.7, 38.4)	8.8 (-9.9, 31.3)	249	25.6 (14.5, 41.1)	4.9 (-9.6, 21.6)	0.772
Nutrition	98	40.6 (29.4, 51.8)	69.2 (41.2, 102.8)	238	36.7 (24.8, 52.0)	51.3 (34.0, 70.9)	0.305
WSH+N	108	42.0 (30.3, 58.8)	81.2 (48.8, 120.8)	264	38.2 (25.1, 52.2)	57.0 (38.4, 78.2)	0.194
WSH+N vs WSH			66.6 (44.1, 92.7)			49.8 (31.5, 70.6)	0.298
WSH+N vs N			7.1 (-7.4, 23.9)			3.8 (-6.6, 15.2)	0.701
**sTfR (mg/L)[1]**							
Control	82	7.3 (6.3, 8.7)		228	7.1 (6.1, 8.5)		
WSH	121	7.2 (6.2, 8.5)	-0.9 (-10.1, 9.2)	249	7.1 (6.0, 8.4)	-1.8 (-9.3, 6.3)	0.879
Nutrition	98	6.5 (5.9, 7.3)	-14.7 (-21.5, -7.2)	238	6.5 (5.9, 7.6)	-11.4 (-18.0, -4.4)	0.462
WSH+N	108	6.6 (6.0, 7.4)	-12.8 (-19.5, -5.6)	264	6.8 (6.0, 7.7)	-10.4 (-16.3, -4.0)	0.565
WSH+N vs WSH			-12.0 (-17.9, -5.8)			-8.7 (-12.9, -4.3)	0.412
WSH+N vs N			2.2 (-2.8, 7.4)			1.2 (-3.9, 6.6)	0.774
**Hepcidin (ng/mL)[1]**							
Control	46	13.6 (6.3, 26.0)		110	13.9 (5.7, 23.0)		
WSH	73	10.9 (6.2, 22.1)	4.7 (-33.1, 63.9)	144	14.5 (6.4, 27.9)	-10.0 (-36.3, 27.2)	0.572
Nutrition	50	18.3 (8.9, 37.0)	66.2 (4.3, 164.8)	128	16.7 (9.8, 26.9)	42.3 (10.2, 83.7)	0.515
WSH+N	52	18.5 (13.8, 33.4)	100.2 (35.0, 196.9)	129	19.1 (10.9, 29.9)	51.8 (17.6, 95.8)	0.199
WSH+N vs WSH			91.2 (41.3, 158.6)			68.6 (16.3, 144.3)	0.638
WSH+N vs N			20.5 (-14.8, 70.3)			6.6 (-12.5, 29.9)	0.544
**B12 (pmol/L)[1]**							
Control	82	274.4 (218.4, 325.6)		222	317.2 (246.5, 417.5)		
WSH	118	318.4 (254.2, 427.4)	16.0 (5.7, 27.2)	242	310.6 (247.9, 423.9)	1.3 (-6.7, 10.0)	0.014
Nutrition	97	354.4 (278.9, 469.6)	29.5 (17.5, 42.8)	235	343.9 (271.4, 416.9)	5.4 (-3.3, 14.8)	0.001
WSH+N	105	345.6 (261.0, 440.8)	22.6 (11.8, 34.3)	258	340.9 (248.0, 460.0)	8.2 (-0.2, 17.2)	0.026
WSH+N vs WSH			5.7 (-5.4, 18.0)			6.8 (0.0, 14.0)	0.880
WSH+N vs N			-5.4 (-15.2, 5.6)			2.6 (-4.9, 10.7)	0.203

[1]Log transformed for analysis.

References

1. Institute of Medicine. Dietary Reference Intakes: The essential guide to nutrient requirements. Washington, DC: National Academies Press, 2006.
2. WHO, FAO. Vitamin and Mineral Requirements in Human Nutrition. Second Edition ed. Geneva, Switzerland: World Health Organization, 2004.
3. Ballard T, Coates J, Swindale A, Deitchler M. Household Hunger Scale: Indicator Definition and Measurement Guide. Washington, DC: Food and Nutrition Technical Assistance (FANTA-II) Project, FHI 360, 2011.
4. Coates J, Swindale A, Bilinsky P. Household Food Insecurity Access Scale (HFIAS) for Measurement of Food Access: Indicator Guide. Washington, DC: Academy for Educational Development, Food and Nutrition Technical Assistance Project (FANTA), 2007.
